# Revealing the significance of tissue-resident memory T cells in lung adenocarcinoma through bioinformatic analysis and experimental validation

**DOI:** 10.3389/fimmu.2025.1600863

**Published:** 2025-06-26

**Authors:** Zhuoqi Li, Mei Tian, Yuanhui Yang, Yuanyuan Wang, Lu Zhang, Fujing Huang, Xiao Wen, Xiaoshu Yin, Xiaoyan Lin, Yuan Tian

**Affiliations:** ^1^ Department of Radiotherapy Oncology, Affiliated Hospital of Shandong University of Traditional Chinese Medicine, Jinan, China; ^2^ Department of Respiratory and Critical Care Medicine, Affiliated Hospital of Shandong University of Traditional Chinese Medicine, Jinan, China; ^3^ Department of Pathology, Shandong Provincial Hospital, Shandong University, Jinan, China; ^4^ Department of Oncology, The Second Affiliated Hospital of Shandong University of Traditional Chinese Medicine, Jinan, Shandong, China; ^5^ Department of Pathology, Shandong Provincial Hospital Affiliated to Shandong First Medical University, Jinan, China

**Keywords:** T_RM_ cells, LUAD, prognostic signature, immunotherapeutic outcomes, TYMS

## Abstract

**Purpose:**

To investigate the functions of lung T_RM_ cells in the development and treatment of lung adenocarcinoma (LUAD).

**Methods:**

R-language bioinformatics analysis was applied to obtain differentially expressed (DE) lung T_RM_ cell-specific genes and a related prognostic signature, which were further validated using external datasets, immunohistochemical staining images, and biological experiments.

**Results:**

A total of 130 DE lung T_RM_ cell-specific genes were identified, 14 of which were involved in the prognostic signature, including *SLC16A3*, *ARHGAP11A*, *PTTG1*, *DTL*, *GPRIN1*, *EXO1*, *GAPDH*, *TYMS*, *DAPK2*, *CCL20*, *HLA-DQA1*, *ADAM12*, *ALOX5AP* and *OASL*. The signature was efficient and robust in predicting the overall survival and anti-PD-1/PD-L1 immunotherapeutic outcomes of patients with LUAD. The AUCs for predicting the 1-, 3-, and 5-year survival rates were 0.688, 0.698, and 0.648, respectively, in the training cohort, and were 0.867, 0.662, and 0.672, respectively, in the validation cohort. The signature also had predictive value for the sensitivity of patients to chemical drugs. *TYMS* was a hub gene in the prognostic signature, and was strongly associated with LUAD progression and cell proliferation in the experimental validation.

**Conclusions:**

The lung T_RM_ cell-related prognostic signature is an effective tool for predicting the prognosis and therapeutic outcomes of patients with LUAD.

## 1 Introduction

Tissue-resident memory T (T_RM_) cells are a special subpopulation of memory T cells that were recently discovered to reside in non-lymphoid tissues without entering the bloodstream (1). T_RM_ cells can reside in a wide range of tissues, including epithelial barrier tissues, such as the lungs, gastrointestinal tract, and skin, as well as non-barrier tissues, such as the brain, kidneys, and joints (2–4). T_RM_ cells are also found in many types of tumor tissues, such as lung cancer, breast cancer, intestinal cancer, ovarian cancer, and melanoma tissues, and they play important roles in anti-tumoral immunology (5–9). The infiltration of T_RM_ cells is a favorable factor for the prognosis of cancer patients, and the abundance of CD103^+^CD8^+^T cells in tumor tissues is correlated with increased disease-free survival and overall survival in patients with lung, breast, endometrial, and ovarian cancers (10). However, the underlying mechanisms are not well understood.

Recently, it has been demonstrated that T_RM_ cells in different organs and tissue sites are specific and play different roles (11). Through the integration of single-cell protein and transcriptome analyses, T_RM_ cell-specific genes associated with major barrier sites in the human body, such as the lungs, skin, and jejunum, were identified, and these T_RM_ cell-specific genes were closely related to the specific functions of each organ (11). Whether the T_RM_ cell-specific genes of an organ can regulate T_RM_ cells to exert specific immune responses against tumors at that tissue site is a question that needs to be addressed.

Lung cancer remains one of the most prevalent cancers worldwide and causes the most cancer-related deaths (12). Lung adenocarcinoma (LUAD) is a type of non-small-cell lung cancer that accounts for the highest percentage of lung cancer cases. LUAD is often accompanied by both genomic and morphological abnormalities. However, its pathogenesis is not well understood, and more effective treatments are currently being explored (13, 14). The tumor immune microenvironment (TME) is an important cause of heterogeneity in lung adenocarcinoma and can influence disease progression and the response to therapy (15). T_RM_ cells are important components of the tumor microenvironment, and the infiltration of CD103^+^CD8^+^T_RM_ cells into the tumor microenvironment has been reported to be a favorable prognostic factor for patients with LUAD (16–20). However, the underlying mechanisms have not yet been elucidated.

Although the role of T_RM_ cells in lung cancer immunomodulation and immunotherapy has been partially reported in some studies, research methods have been limited mostly to experiments on cells and animals, and there are few reports on the use of bioinformatics methods to explore novel biomarkers that can regulate the functions of T_RM_ cells and potentially become predictive and therapeutic biomarkers. In this study, the lung T_RM_ cell-specific genes identified in previous studies were subjected to bioinformatics analysis in many LUAD samples to identify novel potential biomarkers related to the prognosis, TME landscape, and immunotherapy of patients with LUAD. The functions of key genes in LUAD were validated using *in vitro* experiments. These findings may help elucidate the roles of T_RM_ cells in LUAD and identify novel biomarkers for personalized prediction and treatment of LUAD.

## 2 Materials and methods

### 2.1 Data collection and preprocessing

The R package “TCGAbiolinks” was used to download the log-transformed FPKM expression profiles and clinical information from the TCGA-LUAD dataset. A total of 497 tumor samples with both expression data and survival information were retained for the construction of the prognostic signature. The GSE41271 and GSE42127 bulk expression datasets were downloaded from the Gene Expression Omnibus (GEO) database (https://www.ncbi.nlm.nih.gov/geo/) and were used to validate the prognostic signature. The data processing standard of the GEO bulk expression dataset was as follows: the probes were converted to gene symbols according to the probe correspondence with the platform. If one probe corresponded to multiple genes, the probe was removed, and if multiple probes corresponded to the same symbol, the median value was taken. Single-cell RNA-seq data were obtained from the GSE131907 dataset of the GEO database. Fifteen primary LUAD samples from the GSE131907 dataset were used for the analyses. The clinical and transcriptomic data of the GSE126044 and GSE135222 cohorts, in which NSCLC patients were treated with the PD-1/PD-L1 blockade, were downloaded from the GEO database and used to evaluate the predictive efficacy of the prognostic signature. A total of 480 lung T_RM_ cell-specific genes were obtained from a previous publication (11).

### 2.2 Identification of differentially expressed lung T_RM_ cell-related genes

The R package “limma” was used to identify the differentially expressed genes (DEGs) between LUAD and adjacent normal tissues, with thresholds set at |log2FC|≥1 and FDR<0.05. The DEGs intersecting with the lung T_RM_ cell-specific genes were regarded as differentially expressed (DE) T_RM_ cell-related genes and were chosen for subsequent analysis.

### 2.3 Construction of protein-protein interaction networks (PPIs) for DE lung T_RM_ cell-related genes

The interactive relationships of the lung T_RM_ cell-related genes were acquired from the STRING database (https://www.string-db.org/), and a protein-protein interaction (PPI) was constructed based on this information.

### 2.4 Functional enrichment of the DE lung T_RM_ cell-specific genes

The R package “clusterProfiler” was applied for the functional annotation of the DE lung T_RM_ cell-related genes, with the p-value cutoff set at 0.05. Functional enrichment analysis was performed to predict the potential biological functions of these genes.

### 2.5 Construction of the lung T_RM_-related prognostic signature

Univariate Cox regression analysis was used to determine the hazard ratios (HR) and prognostic significance. Genes with p values < 0.05 were prognosis-associated genes. Least Absolute Shrinkage and Selection Operator (LASSO) regression analysis was applied to further identify key prognostic factors, and a risk score model for predicting survival was constructed by weighting the expression of each key prognostic gene with LASSO regression coefficients (“exp” represents the expression level of the genes, and “coef” represents the Cox regression coefficient):


Risk score=∑exp∗coef

The patients were divided into high-risk and low-risk groups based on the median risk score. The “Kaplan-Meier” method was used to generate survival curves for the prognostic analysis, and the “log-rank” test was used to evaluate the significance of the differences in overall survival between groups. The receiver operating characteristic (ROC) curve was used to assess the predictive efficacy of the prognostic models. The R package “timeROC” was used to visualize the “area under the curve” (AUC). Univariate and multivariate Cox regression analyses were performed to evaluate the independent predictive value of the prognostic model.

### 2.6 Evaluation of the TME landscape

The “ESTIMATE” algorithm was used to calculate the immunity score, stroma score, and tumor purity for each tumor sample, and then the “Wilcoxon” test was subsequently used to compare the differences in the immunity score, stroma score, and tumor purity among different subgroups of samples. The correlations between the risk score and the immunity score, stroma score, and tumor purity were calculated using Spearman analysis. Single-sample gene set enrichment analysis (ssGSEA) was used to evaluate the relative abundance of each infiltrating cell in the TME. The gene sets of the 28 types of immune cells used in the analysis were obtained from a previous publication (21). The R packages “GSVA” and “GSEABase” were used to compare the differences in biological pathways and immune functions.

### 2.7 Prediction of drug sensitivity

The “calcPhenotype” function of the R package “oncoPredict” was used to assess the IC50 values of the samples for the drugs. The correlation coefficients between the risk score, the expression of genes included in the prognostic model, and the drug IC50 values were calculated using Spearman analysis.

### 2.8 Quality control for the scRNA-seq data

The R package “Seurat” (version 4.1.0) was used for quality control of the scRNA-seq data. To exclude some low-quality cells and genes expressed at low levels, we set the thresholds as follows (1): each gene was expressed in at least three cells (2); the number of features per cell was between 500 and 6000, and the number of counts per cell was between 1000 and 20,000; and (3) the number of mitochondrial and erythrocyte genes was less than 20% of the total number of genes in each cell. Next, the “NormalizeData” function was used for normalization, and the “FindVariableFeatures” function was used to identify highly variable genes on the basis of their average expression values (greater than 0.1 and less than 3) and dispersion (greater than 0.5). The R package “Harmony” was used to perform batch correction between the samples to avoid batch effects interfering with downstream analysis. The data were then scale transformed and downscaled via principal component analysis (PCA), and the top 50 principal components were selected for downstream analysis and visualized via the “RunTSNE” function.

### 2.9 Identification of the subtypes of malignant tumor cells

Malignant cells in which at least two model genes were detected were selected for subsequent analysis. After standardization, normalization, identification of highly variable genes, removal of batch effects and PCA, the first 50 principal components were selected at a resolution of 0.1. Three subtypes of tumor cells were subsequently identified by clustering and grouping again. The marker genes of each subtype of tumor cells were identified via the “FindAllMarkers” function (avg_log2fc > 0.25, p_val_adj < 0.05). The CellScore was calculated based on the genes included in the prognostic model via the “AddModuleScore” function of the “Seurat” package. The malignant cells were divided into high and low groups based on the median cell score.

### 2.10 Trajectory analysis and cellular communication analysis

The R package “monocle2” was used to conduct the trajectory analysis of the tumor cells. Different states reflect the internal transformation of tumor cells. The R package “CellChat” was used to analyze the communication between tumor cells and other cells.

### 2.11 Validation of the expression levels of genes via immunohistochemical staining images

The expression levels of the genes included in the lung T_RM_ cell-related prognostic model were validated at the protein level using immunohistochemical staining images from the Human Protein Atlas database (https://www.proteinatlas.org/). The staining intensity levels of each gene in normal lung tissues and LUAD tissues were observed and compared.

### 2.12 Clinical sample collection and immunohistochemistry

Lung adenocarcinoma samples were collected from the pathology department of Shandong Provincial Hospital from 2017 to 2021. Written informed consent was obtained from all participants. Tumor tissues were obtained from excised biopsies, fixed in formalin and embedded in paraffin (FFPE) for histological evaluation. After paraffin wax removal and rehydration, the sections were placed in citrate antigen retrieval solution and boiled for 15 minutes for antigen retrieval. An endogenous peroxidase blocker was then added to block the endogenous peroxidase activity in the sections. After incubation at room temperature for 30 min, 50 µL of goat serum working solution was added to each sample, which was subsequently incubated at 37°C for 20 min to block nonspecific staining. The sections were subsequently incubated with a primary antibody (rabbit anti-thymidylate synthase antibody, 1:100, ab108995, Abcam) for 1 h at 37°C. After 3 × 5-minute washes with PBS, the sections were incubated with a biotinylated secondary antibody at room temperature for 30 min, followed by subsequent washes (3 × 5 min in PBS). The sections were subsequently dried with absorbent paper and incubated with 50 µL horseradish peroxidase-labeled streptavidin for 20 min at 37°C. The sections were then rinsed with PBS for 3 × 5 min each. After immunostaining, the sections were visualized using an MBMbio Intelligence 400 scanner according to the manufacturer’s protocol. The slides were independently examined by two experienced pathologists according to the WHO criteria. The expression levels of each gene were characterized using a scoring system. The staining intensity was graded into four levels: 0, no positive staining (negative), 1 point for light yellow (weakly positive), 2 points for brownish yellow (positive); and 3, dark brown (strongly positive). The percentage of positive cells was also classified into four levels: 1 point was given when it was ≤25%, 2 points when it ranged from 26% to 50%, 3 points when it was between 51% and 75%, and 4 points when it was >75%. The final scoring results were obtained by multiplying the scores of the above two items. Based on the results, the samples were divided into four grades: negative expression (0 points), low expression (1–4 points), moderate expression (5–8 points) and high expression (9–12 points).

### 2.13 Cell lines and culture

The human cell line, H1395, was purchased from the National Laboratory Cell Resource Sharing Platform (Beijing, China) at the beginning of this study, with STR authentications. H1395 cells were cultured in RPMI 1640 medium supplemented with 10% fetal bovine serum (FBS) and 100 U/mL penicillin/streptomycin (Invitrogen, Carlsbad, CA, USA) at 37°C in a humidified incubator with 5% CO_2_.

### 2.14 siRNA design and transfection

The siRNA oligo sequences (5’-3’) against *TYMS* mRNAs (si-*TYMS*-1#: sense, GGGAUUCUCCACCAGAGAATT; antisense, UUCUCUGGUGGAGAAUCCCTT; si-*TYMS*-2#: sense, CCAACUGCAAAGAGUGAUUTT; antisense, AAUCACUCUUUGCAGUUGGTT) were synthesized by GenePharma Co. (Shanghai, China). H1395 cells were transfected with the siRNAs using Omifection-R (OMIGET, China) siRNA transfection reagent according to the manufacturer’s instructions when the cells reached a confluence of 60–80% confluence. The successful knockdown of *TYMS* expression was confirmed by quantitative RT-PCR (qRT-PCR) and western blotting 48 h post-transfection. Scramble siRNAs (sense: 5’-UUCUCCGAACGUGUCACGUTT-3’; antisense: 5’-ACGUGACACGUUCGGAGAATT-3’) were used as negative controls.

### 2.15 RNA extraction and quantitative real-time PCR

The total RNA of the cell lines was isolated using the Total RNA Isolation kit (TRIcom Reagent) of GenStone Biotech and then reverse-transcribed into cDNA using TransScript First-Strand cDNA Synthesis SuperMix (TransGen Biotech, China) according to the manufacturer’s instructions. Next, qRT-PCR was performed using the FastStart Universal SYBR Green Master (ROX) (Roche, Germany) on an ABI-7500 Fast system (Applied Biosystems). *ALU* was used as the endogenous reference gene for the cultured cell lines. Each sample was analyzed quantitatively in six replicates. The relative expression levels of these genes were determined using the ΔΔCt method. The differences in target gene expression between different groups were analyzed using the Kruskal-Wallis test and plotted using GraphPad Prism 10.1.2. P < 0.05 was considered statistically significant (***indicates p < 0.001). The primer sequences are shown in **Table 1**.

**Table 1:** The sequences of the primers used in the study.

*TYMS*	Sequence (5’ → 3′)
Forward Primer	GTGTGCCTTTCAACATCGCC
Reverse Primer	GGGTTCTCGCTGAAGCTGAAT
*ALU*	Sequence (5’ → 3′)
Forward Primer	GAGGCTGAGGCAGGAGAATCG
Reverse Primer	GTCGCCCAGGCTGGAGTG

### 2.16 Western blotting

Total protein was extracted from cultured cells using RIPA buffer. Primary polyclonal antibodies against *TYMS* (15047-1-AP, ProteinTech) and *β-Actin* (66009-1-Ig, ProteinTech) were used at dilutions of 1:3,000 and 1:20,000, respectively. The signals were visualized using an enhanced chemiluminescence kit (Millipore) and an Alpha Imager system.

### 2.17 Assessment of cell proliferation with IncuCyte

The long-term dynamic proliferation of the H1395 cells was observed using a long-term dynamic observation platform (IncuCyte, Essen, MI, USA). The cells were seeded into 96-well plates (3000 cells per well, six wells per group) and cultured for 120 h to generate proliferation curves. The cells were photographed every 24 h on the platform and analyzed using IncuCyte ZOOM software (Essen, Ann Arbor, MI, USA).

### 2.18 Statistical analysis

All analyses were performed using R software (version 4.4.2). For significance analysis between various values (such as expression levels, infiltration ratios, and various eigenvalues), the Wilcoxon rank-sum test was applied to compare the differences between two groups of samples, and the Kruskal-Wallis test was used to compare the differences between multiple groups of samples. For the plot presentation, ns indicates p > 0.05, * indicates p < 0.05, ** indicates p < 0.01, *** indicates p < 0.001, and **** indicates p < 0.0001. Survival curves for the prognostic analysis were generated using the Kaplan-Meier method, and the significance of the differences was determined using the log-rank test.

## 3 Results

### 3.1 Identification of DE lung T_RM_ cell-specific genes

**Figure 1:**
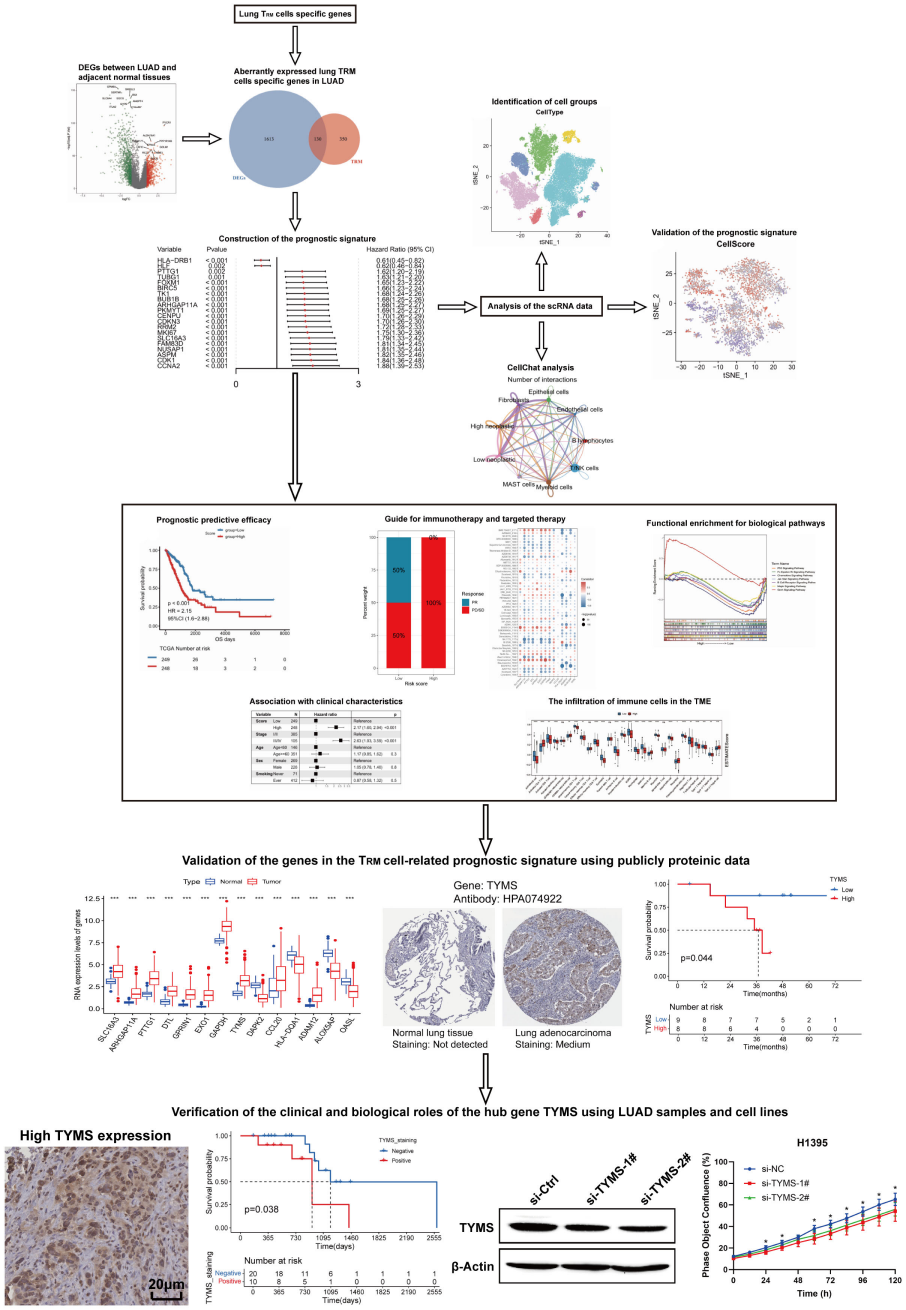
The flow chart of this study.

**Figure 2:**
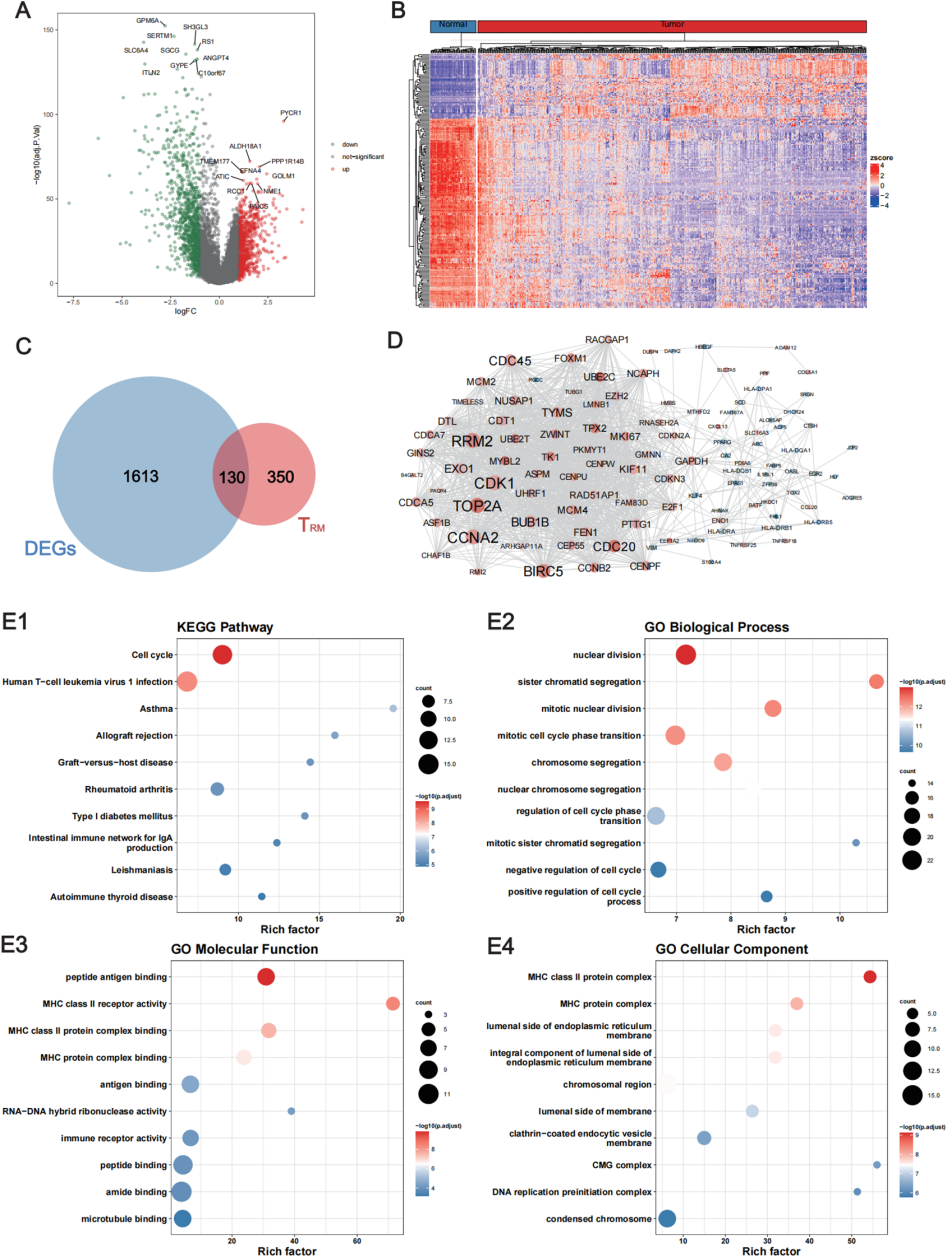
Identification of the lung T_RM_ cell-related genes that were differentially expressed between LUAD and adjacent normal tissues. **(A)** Volcano plot showing the DEGs between LUAD and adjacent normal tissues. The red dots represent the upregulated genes with log2FC ≥ 1 and FDR < 0.05, whereas the green dots represent downregulated genes with log2FC ≤ -1 and FDR < 0.05. **(B)** Heatmap of the DEGs. The upper horizontal axis denotes the cluster analysis of each sample. The blue color indicates adjacent normal tissues, whereas the red color indicates tumor tissues. The left longitudinal axis indicates the cluster analysis of the DEGs. The blue and red blocks represent relatively low and high expression, respectively. **(C)** Venn diagram showing the intersecting genes among the DEGs and the T_RM_ cell-related genes in the lung. **(D)** The PPI network of the intersecting genes among the DEGs and the lung T_RM_ cell-related genes. **(E1)** KEGG pathway, **(E2)** GO biological process, **(E3)** GO molecular function and **(E4)** GO cellular component enrichment analyses of the intersecting genes. FC, fold change; FDR, false discovery rate; DEGs, differentially expressed genes.

A flow chart of the study is shown in **Figure 1**. To assess whether the expression of lung T_RM_-cell-specific genes affects tumorigenesis and tumor progression in LUAD, differential expression analysis was performed in LUAD and adjacent normal tissues. First, 1002 downregulated genes and 741 upregulated genes in tumor tissues were screened (**Figures 2A, B**; **Supplementary Table 1**), including 130 lung T_RM_ cell-specific genes (**Figure 2C**; **Supplementary Table 2**). Protein-protein interaction network (PPI) analysis results revealed extensive interactions among the DE lung T_RM_ cell-specific genes, and the node connectivity of the *RRM2*, *CDK1*, *CCNA2* and *EXO1* genes was relatively high, which may indicate that these genes play a dominant role in the regulatory network (**Figure 2D**).

The results of functional enrichment analysis revealed that the DE lung T_RM_ cell-specific genes were significantly enriched in biological processes such as cell cycle regulation and chromosome segregation and were significantly associated with functions such as MHC class II molecule receptor activity, antigen binding, and immune receptor activity (**Figures 2E1–E4**), suggesting that these genes are related to T_RM_ cells.

### 3.2 Construction and validation of the lung T_RM_-related prognostic signature

**Figure 3:**
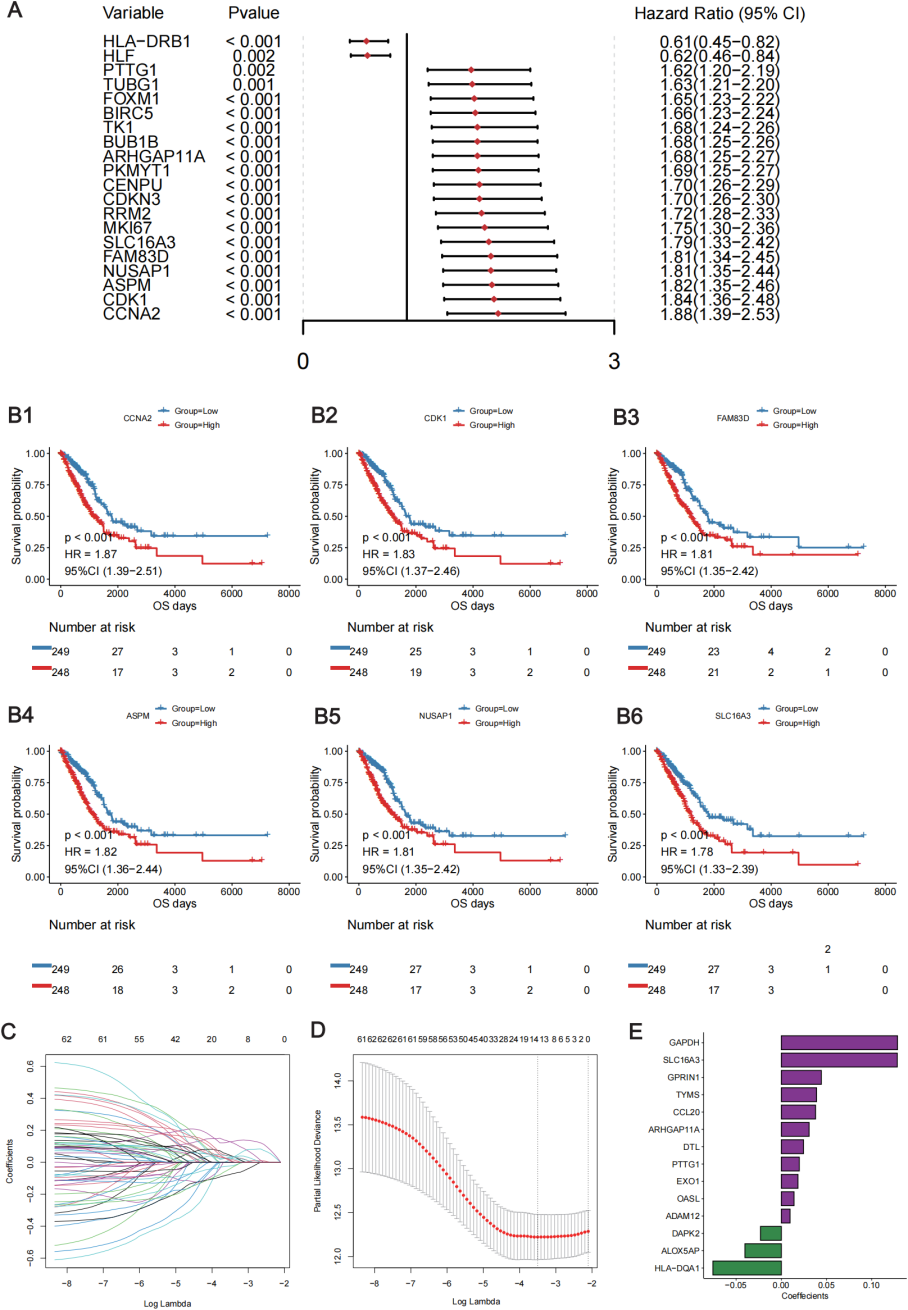
Construction of the lung T_RM_ cell-related prognostic model. **(A)** Forest plot showing the top 20 lung T_RM_ cell-related prognostic genes identified via univariate Cox regression analysis. The left column of each panel shows the p value of each gene, and the right column shows the corresponding forest plot. **(B)** The KM survival curves of the top 6 prognostic genes in the univariate Cox regression analysis: *CCNA2* **(B1)**, *CDK1* **(B2)**, *FAM83D* **(B3)**, *ASPM* **(B4)**, *NUSAP1* **(B5)** and *SLC16A3* **(B6)**. The abscissa axis shows the survival time, whereas the ordinate axis shows the survival probability. The blue color represents low expression, whereas the red color represents high expression of each gene. The risk table is presented under the KM survival curves of each gene. **(C)** Scatter plot showing the trajectory of each independent variable. The abscissa axis represents the log value of the independent variable lambda. The vertical axis indicates the coefficient of the independent variable. **(D)** Dynamic process diagram of variables screened by LASSO regression analysis and selection process diagram of the cross-validation parameter lambda. **(E)** Coefficient of each gene included in the prognostic model.

To investigate the clinical value of the DE lung T_RM_ cell-specific genes, a lung T_RM_ cell-related prognostic signature was constructed and validated. First, a univariate Cox regression analysis was performed. There were 62 genes associated with overall patient survival, and the top 20 genes with the greatest significance are shown in **Figure 3A**; **Supplementary Table 3**. The KM curves of the top six genes with the lowest p-values are presented in (**Figures 3B1–B6**). Least absolute shrinkage and selection operator (LASSO) regression analysis was subsequently conducted to further investigate the clinical significance of these genes. The trajectory of each independent variable was obtained (**Figure 3C**), and as the lambda gradually increased, the number of independent variable coefficients gradually decreased to zero (**Figure 3C**). Ten-fold cross-validation was used to build the model, and the confidence intervals for each lambda value are shown in **Figure 3D**. Fourteen genes were identified when the model was optimized. Therefore, we selected the 14 genes for the subsequent analyses and constructed a risk score model based on their coefficients and expression levels of the 14 genes (**Figure 3E**). The formula for calculating the risk-score model is as follows:

Score = *SLC16A3* * (0.128) + *ARHGAP11A* * (0.031) + *PTTG1* * (0.020) +*DTL* * (0.025) + *GPRIN1* * (0.044) + *EXO1* * (0.018) + *GAPDH* * (0.128) + *TYMS* * (0.039) + *DAPK2* * (-0.023) + *CCL20* * (0.038) + *HLA-DQA1* * (-0.076) +*ADAM12* * (0.010) + *ALOX5AP* * (-0.040) + *OASL* * (0.014).

**Figure 4:**
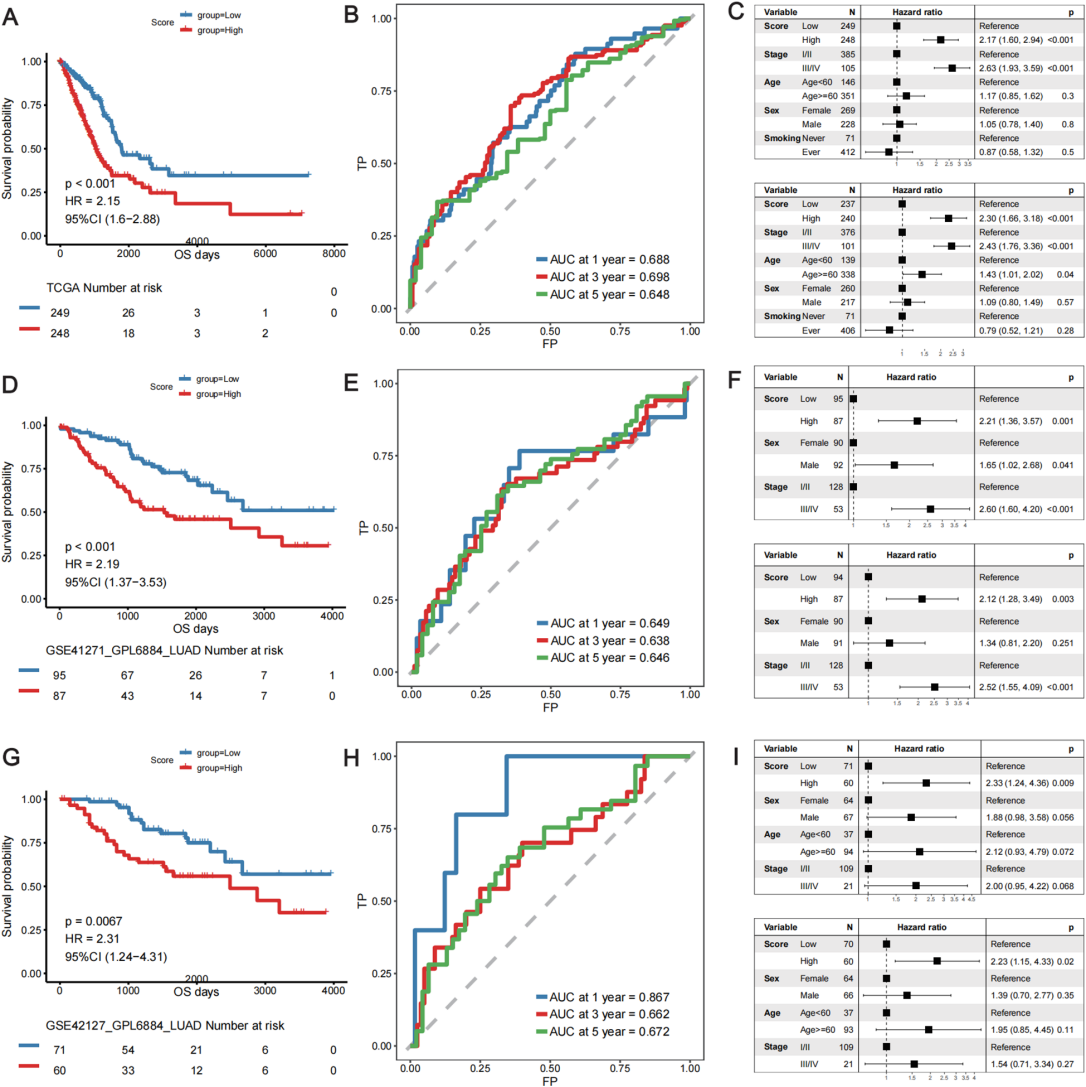
Validation of the predictive efficacy of the lung T_RM_ cell-related prognostic model in the training cohort: **(A–C)** TCGA-LUAD cohort and in the validation cohorts: **(D–F)** GSE41271 and **(G–I)** GSE42127. **(A, D, G)** KM survival curves of patients in the low- and high-risk score groups in the TCGA-LUAD cohort, GSE41271 cohort and GSE42127 cohort, respectively. The blue color represents patients in the low-risk score group, whereas the red color represents patients in the high-risk score group. The risk table is presented under the KM survival curves of each gene. **(B, E, H)** ROC curves for predicting the 1-, 3-, and 5-year survival of patients according to the risk score in the TCGA-LUAD cohort, GSE41271 cohort and GSE42127 cohort, respectively. The abscissa axis represents specificity, and the vertical axis represents sensitivity. Different colors represent different predictive times. **(C, F, I)** Univariate and multivariate Cox regression analyses of the prognostic model in the TCGA-LUAD cohort, GSE41271 cohort and GSE42127 cohort, respectively. The upper forest plot in each panel is the result of univariate Cox regression analysis, whereas the lower plot is the result of multivariate Cox regression analysis. In each forest plot, the variables are listed on the left of each panel. The hazard ratio of each variable and the corresponding forest plot are in the middle of each panel. The p values of the corresponding variables are shown on the right.

Using the 14-gene risk score model, the samples in the TCGA-LUAD training cohort were divided into high- and low-risk groups according to the median risk score. Overall survival analysis revealed that the OS of patients in the high-risk group was significantly lower than that of patients in the low-risk group in both the training cohort (TCGA-LUAD) (**Figure 4A**) and the two validation cohorts: GSE41271 (**Figure 4D**) and GSE42127 (**Figure 4G**). The ROC curve revealed that the AUCs of the patients at 1, 3, and 5 years were relatively high (0.688, 0.698, and 0.648, respectively) in the training cohort (**Figure 4B**). The AUCs of patients at 1, 3, and 5 years were 0.649, 0.638, and 0.646, respectively, in validation cohort GSE41271 (**Figure 4E**). The AUCs of the patients at 1, 3, and 5 years were 0.867, 0.662, and 0.672, respectively, in validation cohort GSE42127 (**Figure 4H**). To test whether the risk score model was an independent prognostic factor for LUAD patients, we performed univariate and multivariate Cox regression analyses via the “coxph()” function in the R package “survival”. In all the training and validation cohorts, the risk score was an independent prognostic factor among other clinical features, such as age, sex, and tumor stage (**Figures 4C, F, I**). These results demonstrated that the 14-gene prognostic signature based on the DE lung T_RM_ cell-specific genes had strong prognostic efficacy with high robustness and generalizability.

### 3.3 Association between the T_RM_ cell-related prognostic signature and clinicopathologic features

The associations between the T_RM_-related prognostic signature and patients’ clinicopathological features were further analyzed. The results revealed that the proportions of patients aged <60 years, with advanced-stage disease, and with a history of smoking was significantly greater in the high-risk score group than in the low-risk score group (p < 0.05). The proportions of patients of different sexes, *ALK* rearrangements, *EGFR* mutations, and *KRAS* mutations did not significantly differ between the high- and low-risk score groups (**Supplementary Figure 1**). Patients aged <60 years, with advanced-stage disease, male sex, and a history of smoking had significantly higher risk scores than the other groups of patients, and there was no significant difference in the risk scores for patients with *EGFR* mutations or *KRAS* mutations (**Supplementary Figure 2**).

### 3.4 Depiction of the TME landscape via the prognostic signature

**Figure 5:**
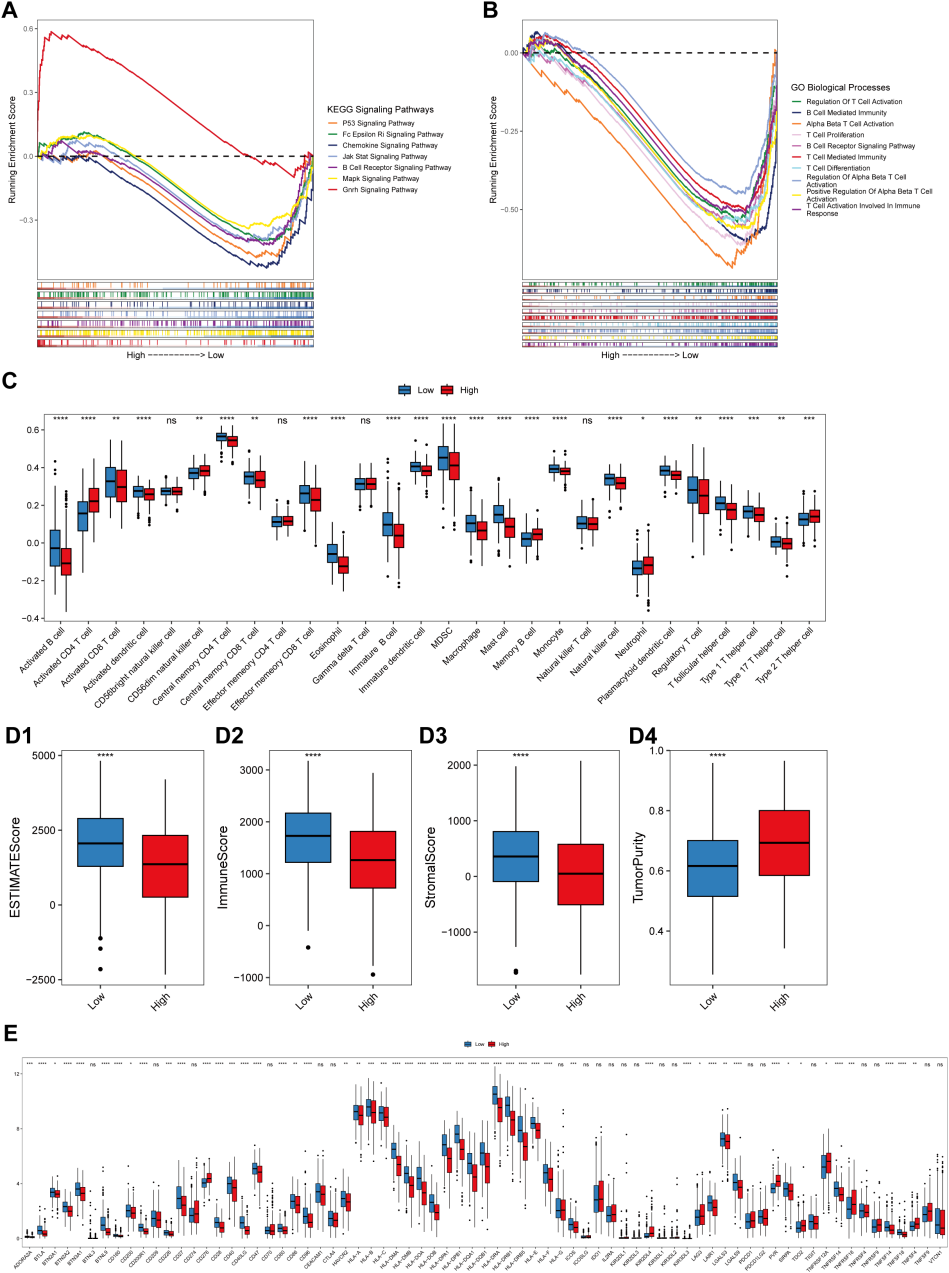
Correlation between the risk score and the immune landscape. **(A)** Pathways that are activated in different risk score groups according to KEGG GSEA enrichment analysis of bulk RNA-seq data. **(B)** Pathways that were activated in different risk score groups according to the GO-BP enrichment analysis of the bulk RNA-seq data. The abscissa axis represents the ranked gene list according to their expression levels in the two groups. The vertical axis represents the running enrichment score. Curves of different colors represent different pathways. **(C)** Relative abundances of the 28 types of immune cells in the low-risk score and high-risk score groups. The abscissa axis represents the names of the immune cells. The vertical axis represents the infiltration fraction. **(D)** Box plots showing the ESTIMATE score **(D1)**, immune score **(D2)**, stromal score **(D3)** and tumor purity **(D4)** in the low- and high-risk score groups. **(E)** Expression levels of immune checkpoint genes in the low-risk score and high-risk score groups. The abscissa axis shows the gene names, and the vertical axis shows the relative expression levels of these genes.

To further explore the functions of lung-specific T_RM_ cells in the TME of LUAD, gene set enrichment analysis (GSEA) and immune cell infiltration analysis were conducted in the high- and low-risk score groups of patients. The results revealed that signaling pathways, such as P53, B-cell receptor, and MAPK, were significantly activated in the low-risk score group (**Figure 5A**; **Supplementary Table 4**), and immune-related biological processes, such as T-cell activation, proliferation, and B-cell activation, were also significantly activated in the low-risk score group (**Figure 5B**; **Supplementary Table 4**). Further analysis of immune cell infiltration revealed that the infiltration of immune cells, such as activated B cells, activated CD8^+^T cells, central memory CD4^+^T cells, central memory CD8^+^T cells, and effector memory CD8^+^T cells, was significantly greater in the low-risk score group (**Figure 5C**). These results demonstrate that patients in the low-risk score group had stronger antitumor immunity and greater infiltration of T_RM_ cells, which may be the reason for their longer survival time. The ESTIMATE, immunity, and stroma scores were significantly greater in the low-risk score group, whereas the tumor purity was significantly greater in the high-risk score group (**Figures 5D1–D4**). Next, the expression levels of the immune checkpoint genes were compared between the high- and low-risk score groups. The results revealed that the expression levels of several immune checkpoint genes, including *CD276* and *LAG3*, were significantly different between the two groups (**Figure 5E**). These findings suggest the possibility of exploring novel targets for immunotherapy.

### 3.5 Validation of the predictive efficacy of the prognostic model at the single-cell level

A total of 51935 cells, including 4827 B lymphocytes, 635 endothelial cells, 10998 epithelial cells, 1764 fibroblasts, 1735 MAST cells, 9098 myeloid cells, 22878 T/NK cells, and 27578 cells, were detected in the GSE131907 scRNA-seq cohort (**Supplementary Figure 3**). The PCA results revealed that there was a significant batch effect between samples (**Supplementary Figures 4A, B**), and the batch effect between samples was removed via the R package “Harmony” (**Supplementary Figures 4C, D**). The distribution of different cell types was determined via UMAP analysis (**Supplementary Figure 4E**), and heterogeneity in the distribution of cells among the samples was detected (**Supplementary Figure 4F**).

**Figure 6:**
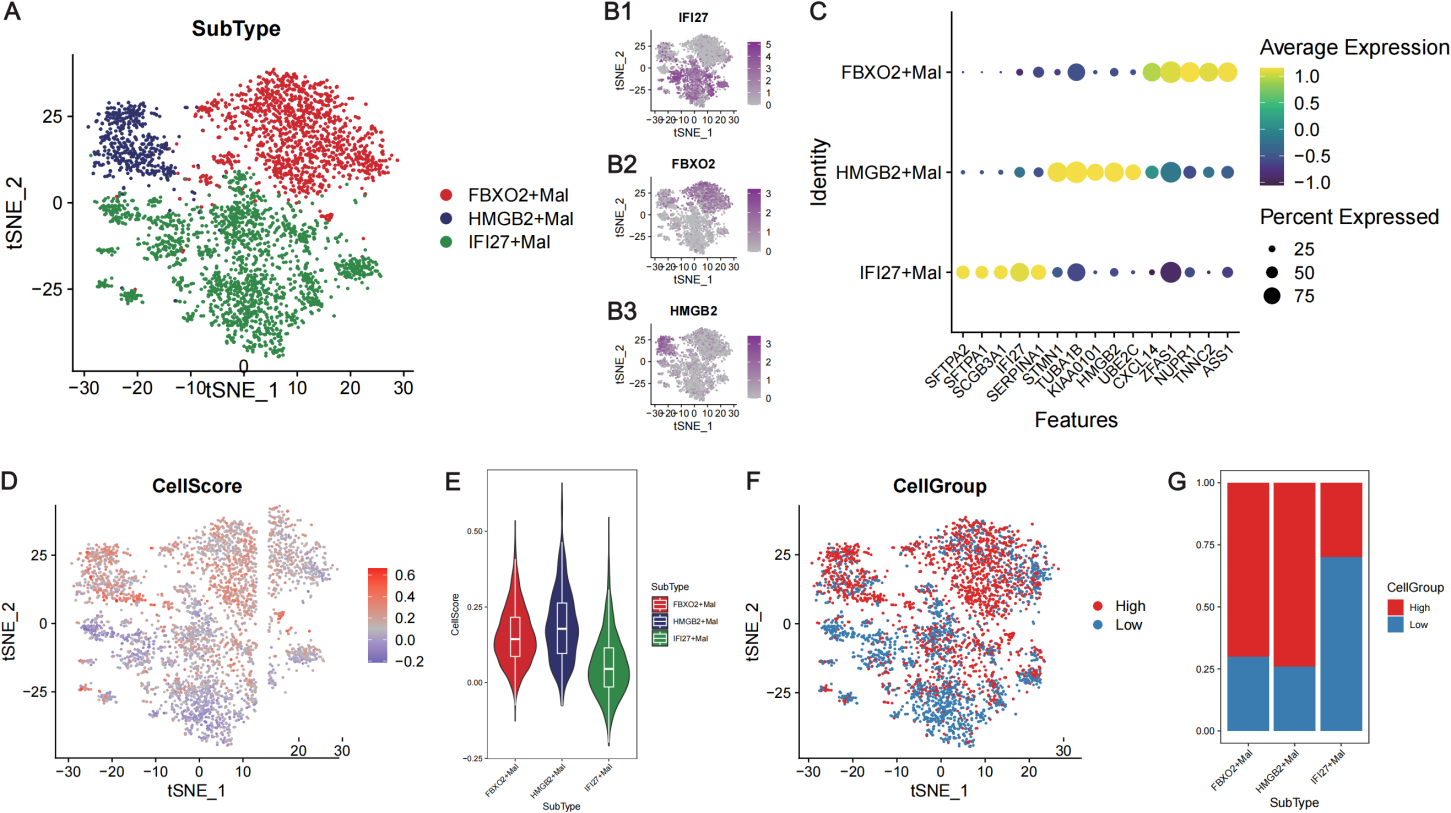
Calculation of the risk score at the single-cell level in LUAD. **(A)** TSNE plot showing the three subtypes of malignant tumor cells. Different colors represent different cell subtypes. **(B)** TSNE plots showing the expression levels of the marker genes in the three subtypes of malignant tumor cells: *IFI27* **(B1)**, *FBXO2* **(B2)** and *HMGB2* **(B3)**. **(C)** Bubble diagram presenting the expression of the top 5 marker genes of the three subtypes of malignant cells. The abscissa axis shows the gene names, and the vertical axis shows the names of the cell subtypes. Yellow indicates high expression, whereas yellow indicates low expression. The bubble size represents the percentage of each gene expressed in each subtype of cell. **(D)** The TSNE plot showing the cell score for each malignant cell. Blue represents a low score, whereas red represents a high score. **(E)** Violin plot showing the cell scores of three subtypes of malignant cells. The abscissa axis shows the cell names, and the vertical axis shows the CellScore. **(F)** The TSNE plot shows that the single cells were divided into high- and low-CellScore groups. **(G)** Proportion of different subtypes of malignant cells in the high- and low-cell groups. The abscissa represents the cell name, and the vertical axis represents the proportion.

A total of 3906 malignant tumor cells with at least two model genes detected were extracted for subsequent analyses. These malignant cells were renormalized and clustered, and three subtypes of malignant cells were identified (**Figures 6A, B1–B3**). These cell subtypes were defined according to the genes that were highly expressed in the clusters, and these three subtypes were named *IFI27* ^+^Mal, *FBXO2* ^+^Mal and *HMGB2* ^+^Mal (**Figures 6A, B1–B3**). With the “FindAllMarkers” function, we identified the marker genes of each cell subtype (**Supplementary Table 5**), and the top 5 marker genes of each cell subtype are shown in **Figure 6C**. *FBXO2* ^+^Mal highly expressed genes such as *CXCL14*, *TNNC2* and *ASS1*, which were significantly enriched in biological processes such as the regulation of the apoptosis signaling pathway and peptidase activity (**Supplementary Figure 5A**). *HMGB2* ^+^Mal highly expressed genes such as *STMN1*, *TUBA1B* and *UBE2C*, which were significantly enriched in biological processes such as the regulation of cell adhesion, leukocyte migration and leukocyte chemotaxis (**Supplementary Figure 5B**). *IFI27* ^+^Mal highly expressed genes such as *SFTPA2*, *SFTPA1* and *SCGB3A1*, which were significantly enriched in biological processes such as the regulation of cell adhesion, leukocyte migration and leukocyte chemotaxis (**Supplementary Figure 5C**).

The CellScore of each malignant tumor cell line was calculated via the “AddModuleScore” function (**Figure 6D**), and the malignant tumor cells were divided into high- and low-CellScore groups (**Figure 6F**). Among the three cell subtypes, *FBXO2* ^+^Mal and *HMGB2* ^+^Mal had higher CellScores (**Figure 6E**), and the CellGroup was high (**Figure 6G**). GSEA of the cells in high- and low-CellScore groups revealed that immune-related biological processes, such as T-cell migration and the B-cell receptor signaling pathway, were also significantly activated in the low-CellScore group (**Supplementary Figure 6**; **Supplementary Table 6**).

**Figure 7:**
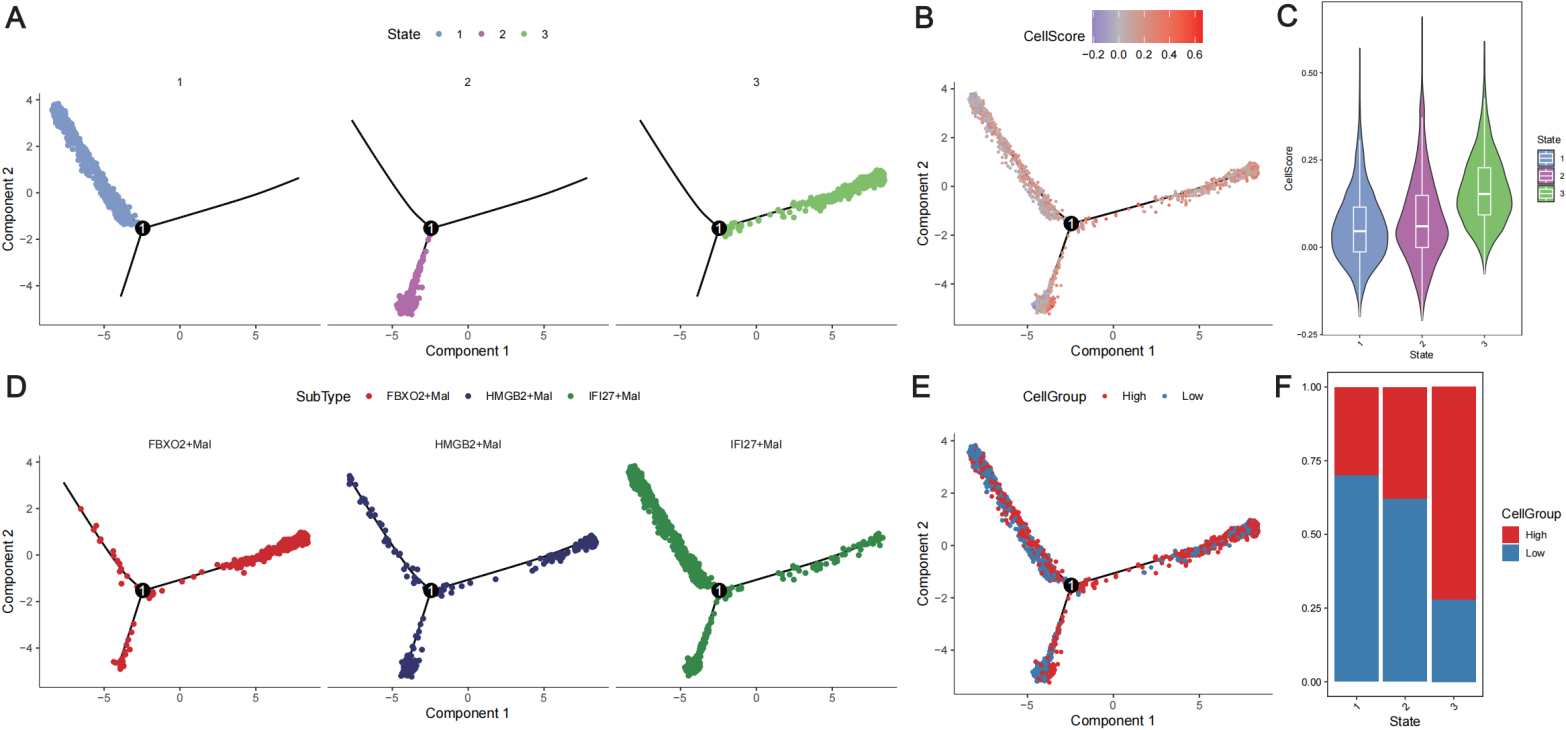
Trajectory analysis of malignant tumor cells. **(A)** Distribution of the three differentiation states of malignant cells. Different colors represent different states. **(B)** Distribution of malignant cells with different cell scores according to the differentiation trajectory. Different colors represent different cell scores. **(C)** Violin plot showing the cell scores in different states of the cell differentiation trajectory. **(D)** Distribution of the three subtypes of cells according to the cell differentiation trajectory. Different colors represent different cell subtypes. **(E)** Distribution of different cell groups according to the cell differentiation trajectory. Red represents a high CellScore, whereas blue represents a low CellScore. **(F)** Distribution of low- and high-cell groups in different cell differentiation states. Red represents the high-CellScore group, whereas blue represents the low-CellScore group.

Trajectory analysis of the extracted malignant epithelial cells revealed three differentiation states (**Figure 7A**). In the trajectory from State1 to State2 cells, the *IFI27* ^+^Mal subpopulation decreased significantly, whereas the *HMGB2* ^+^Mal subpopulation increased significantly (**Figure 7D**). In the State1 to State3 cell trajectories, the proportion of the *FBXO2* ^+^Mal subpopulation increased, but the *HMGB2* ^+^Mal subpopulation also increased (**Figure 7D**). In the trajectory from State1 to State2, there was no significant increase in the CellScore. However, in the trajectory from State1 to State3, there was a significant increase in the CellScore (**Figures 7B, C**) and an increase in the proportion of high-cell groups (**Figures 7E, F**). This suggests that the malignancy of the tumor cells increased from low to high in the trajectory.

To further analyze the differences in physiological activity between high- and low-neoplastic populations, cell-to-cell communication was analyzed via the “CellChat” package. Extensive cellular communication was observed between the cell populations (**Supplementary Figure 7A**). High neoplastic cells were more likely to be outgoing signaling-dominant senders than low neoplastic cells (**Supplementary Figure 7B**), and different cell populations were found to have outgoing signaling patterns in different biological pathways (**Supplementary Figures 7C, D**). Compared with low neoplastic patients, high neoplastic patients exhibited specific cellular communication in the CSF and KIT signaling pathways (**Supplementary Figures 7E, F**).

### 3.6 Lung T_RM_-related prognostic model for the treatment of LUAD

**Figure 8:**
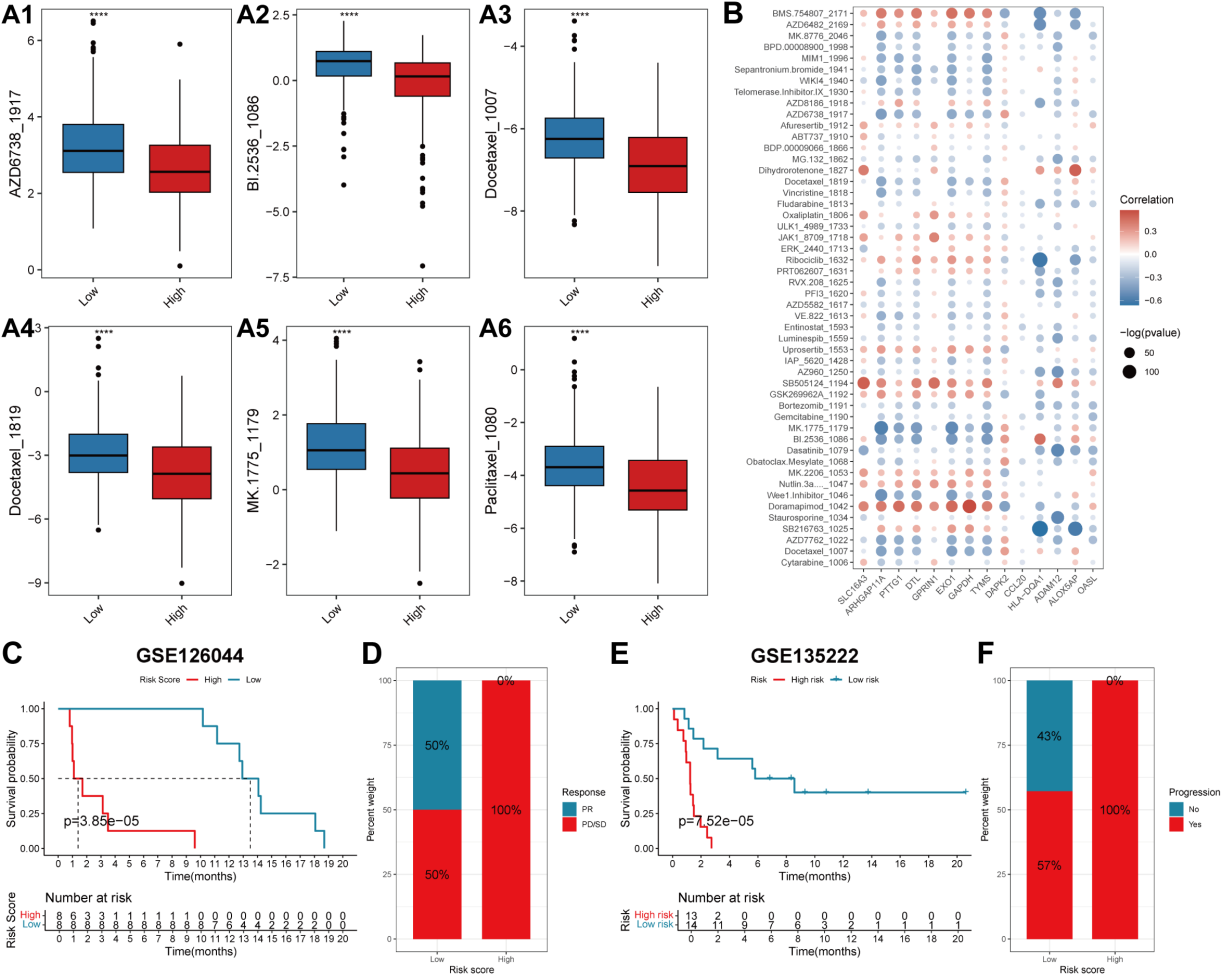
The predictive efficacy of the prognostic model for chemotherapy and immunotherapy. **(A)** Box plots showing the log2(IC50) values of the six drugs that had the highest negative correlation with the risk score in the high- and low-score groups: AZD6738_1917 **(A1)**, BI.2536_1086 **(A2)**, Docetaxel_1007 **(A3)**, Docetaxel_1819 **(A4)**, MK.1775_1179 **(A5)** and Paclitaxel_1080 **(A6)**. In each box plot, the abscissa axis indicates the risk score groups, and the vertical axis indicates the log2(IC50) value of each drug. **(B)** The correlation coefficients between the top 50 drugs that have the highest negative correlation with the risk score and the expression levels of the genes involved in the prognostic signature. The abscissa axis indicates the gene names, and the vertical axis indicates the drug names. Red indicates a positive correlation, whereas blue represents a negative correlation. The sizes of the circles indicate significance (-log(p value)). **(C, E)** KM survival curves of patients in the low- and high-risk score groups in the two immunotherapeutic cohorts GSE126044 and GSE135222. The blue color represents patients in the low-risk score group, whereas the red color represents patients in the high-risk score group. The risk table is presented under the KM survival curves of each gene. **(D)** The proportions of patients who experienced a PR or PD/SD after receiving anti-PD1/PD-L1 immunotherapy in the low- and high-risk score groups. **(F)** The proportions of patients who experienced progressive and no progressive disease after receiving anti-PD1/PD-L1 immunotherapy in the low- and high-risk score groups. PR, partial response; PD, progressive disease; SD, stable disease.

The IC50 values of the drugs in the training cohort were predicted using the R package “oncoPredict” via the use of the drug information from the GDSC database combined with the expression profiles of the training set. Spearman correlation analysis was performed between the prognostic signature and the log2(IC50) value for each drug (**Supplementary Table 7**). Patients in the high-risk group had a poorer prognosis; therefore, the top six drugs with the most significant negative correlations were selected according to the absolute values of the correlation coefficients. The six drugs used were AZD6738_ 1917, BI.2536_1086, docetaxel_1007, docetaxel_1819, MK.1775_179, and paclitaxel_1080 (p<0.05). The log2(IC50) values of these six drugs were lower in the high-risk score group than those in the low-risk score group and had greater sensitivity (**Figures 8A1–A6**). The Spearman correlation coefficients between the risk scores and drug log2 (IC50) values were also calculated, and the top 50 drugs were selected for display, which revealed that there was a correlation between gene expression levels and most of the drug log2 (IC50) values (**Figure 8B**).

To explore the predictive efficacy of the lung T_RM_-related prognostic model for patients receiving anti-PD1/PD-L1 immunotherapy, two immunotherapeutic cohorts, GSE126044 and GSE135222, were used for the prognostic analysis. The results revealed that patients in the low-risk score group had a superior overall survival status compared with patients in the high-risk score group in both cohorts (**Figures 8C, E**). Patients with low-risk scores had a greater response rate (**Figure 8D**) and a greater progression-free rate to anti-PD1/PD-L1 immunotherapy (**Figure 8F**).

### 3.7 Validation of the expression levels of genes in the protein data

**Figure 9:**
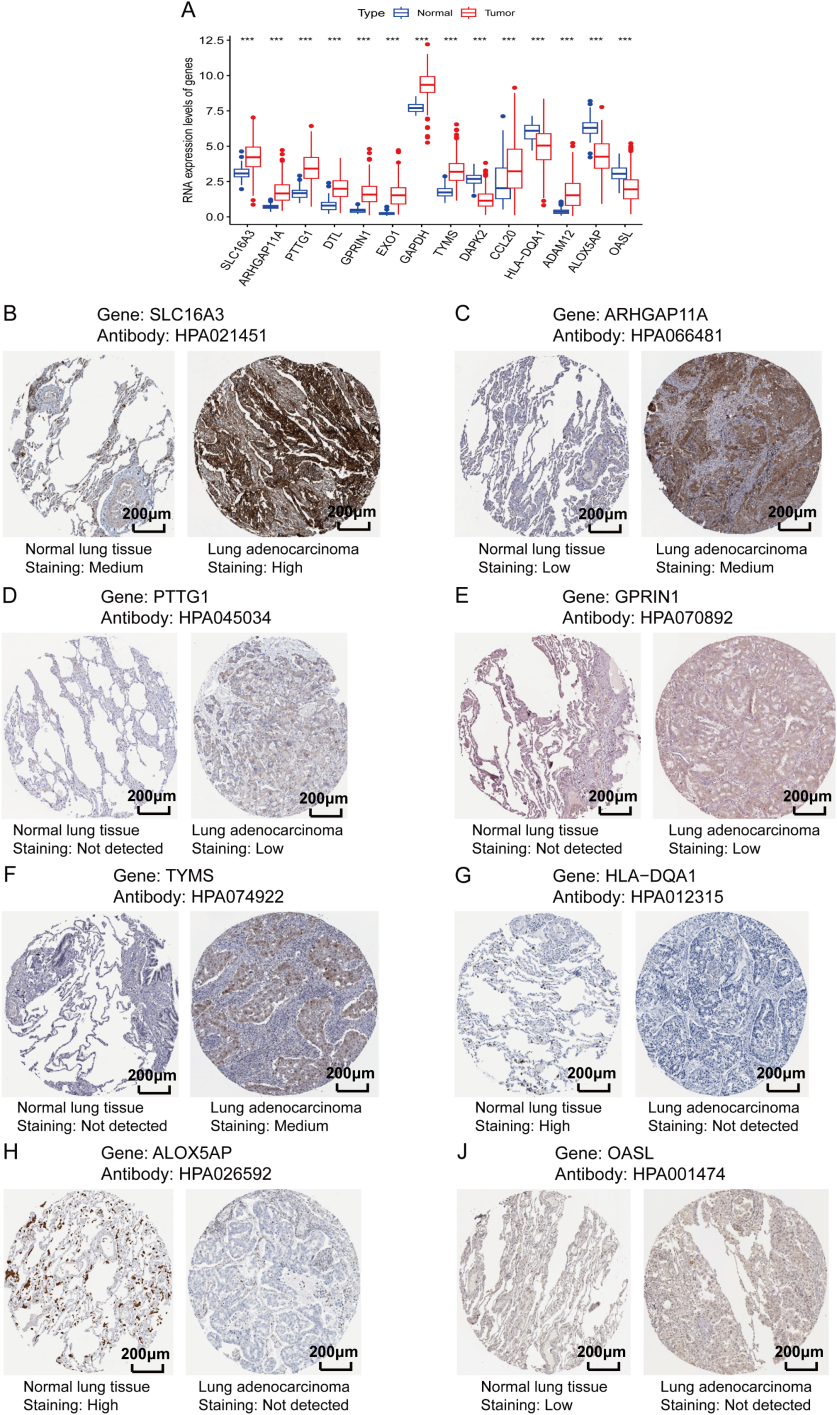
Validation of the protein expression levels of genes involved in the lung T_RM_-related prognostic signature. **(A)** RNA expression levels of the genes included in the prognostic model in LUAD and adjacent normal tissues. The abscissa axis shows the gene names, and the vertical axis shows the RNA expression levels. **(B–J)** Immunohistochemical staining images obtained from the Human Protein Atlas database (https://www.proteinatlas.org/): **(B)** *SLC16A3*, **(C)** *ARHGAP11A*, **(D)** *PTTG1*, **(E)** *GPRIN1*, **(F)** *TYMS*, **(G)** *HLA-DQA1*, **(H)** *ALOX5AP* and **(J)** *OASL*. The names of the genes and antibodies are presented at the top of each panel. The left image of each panel is the adjacent normal tissue, whereas the right image is the LUAD tissue. The staining intensity is labeled under each image.

**Figure 10:**
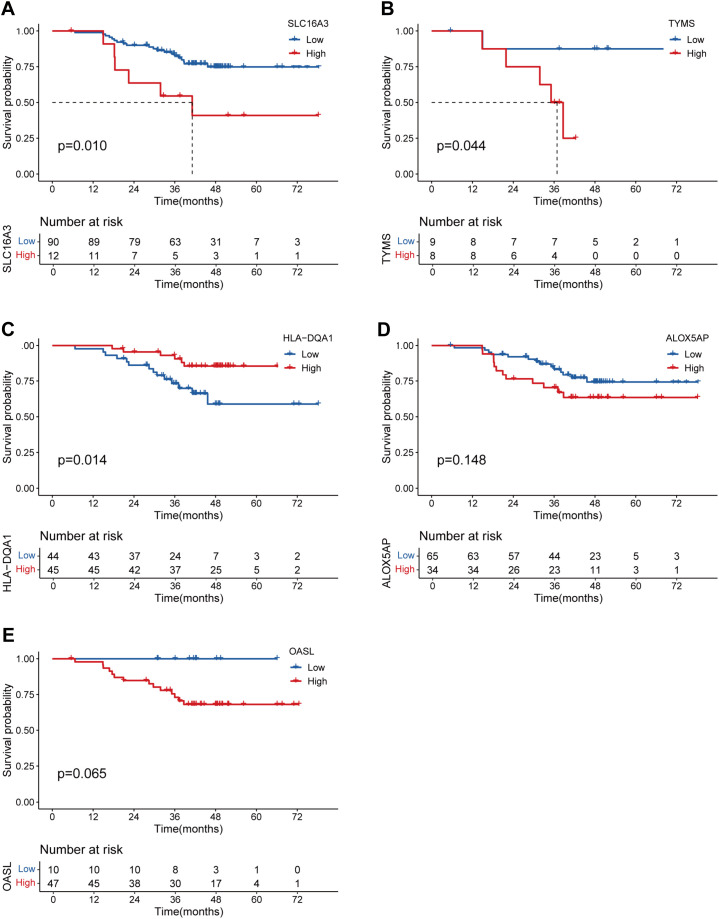
Validation of the prognostic significance of the genes involved in the lung T_RM_-related prognostic signature via proteomic data. K–M curves showing the survival status of LUAD patients with low and high protein expression of **(A)** *SLC16A3*, **(B)** *TYMS*, **(C)** *HLA-DQA1*, **(D)** *ALOX5AP* and **(E)** *OASL*.

To validate whether the protein expression levels of the genes involved in the lung T_RM_ cell-related prognostic model were consistent with the RNA expression levels, immunohistochemical staining images were obtained from the Human Protein Atlas database (https://www.proteinatlas.org/). The results of immunohistochemical staining for *SLC16A3*, *ARHGAP11A*, *PTTG1*, *GPRIN1* and *TYMS* were greater in LUAD tissues than in normal lung tissues, which was consistent with the RNA expression levels of these genes (**Figures 9A–F**). Immunohistochemical staining for *HLA-DQA1*, *ALOX5AP* and *OASL* was lower in LUAD tissues than in normal lung tissues, which was consistent with the RNA expression levels of these genes (**Figures 9A, G–J**). The LUAD proteome expression data and corresponding clinical information were obtained from the supplementary data of the study by Xu et al. (22). Survival analysis based on the proteome data revealed that *SLC16A3*, *TYMS*, *ALOX5AP* and *OASL* were risk factors for patient prognosis, whereas *HLA-DQA1* was a protective factor (**Figures 10A–E**). These findings validated the functions of key genes in the T_RM_ cell-related prognostic signature.

### 3.8 Verification of the clinical and biological roles of the TYMS hub gene

**Figure 11:**
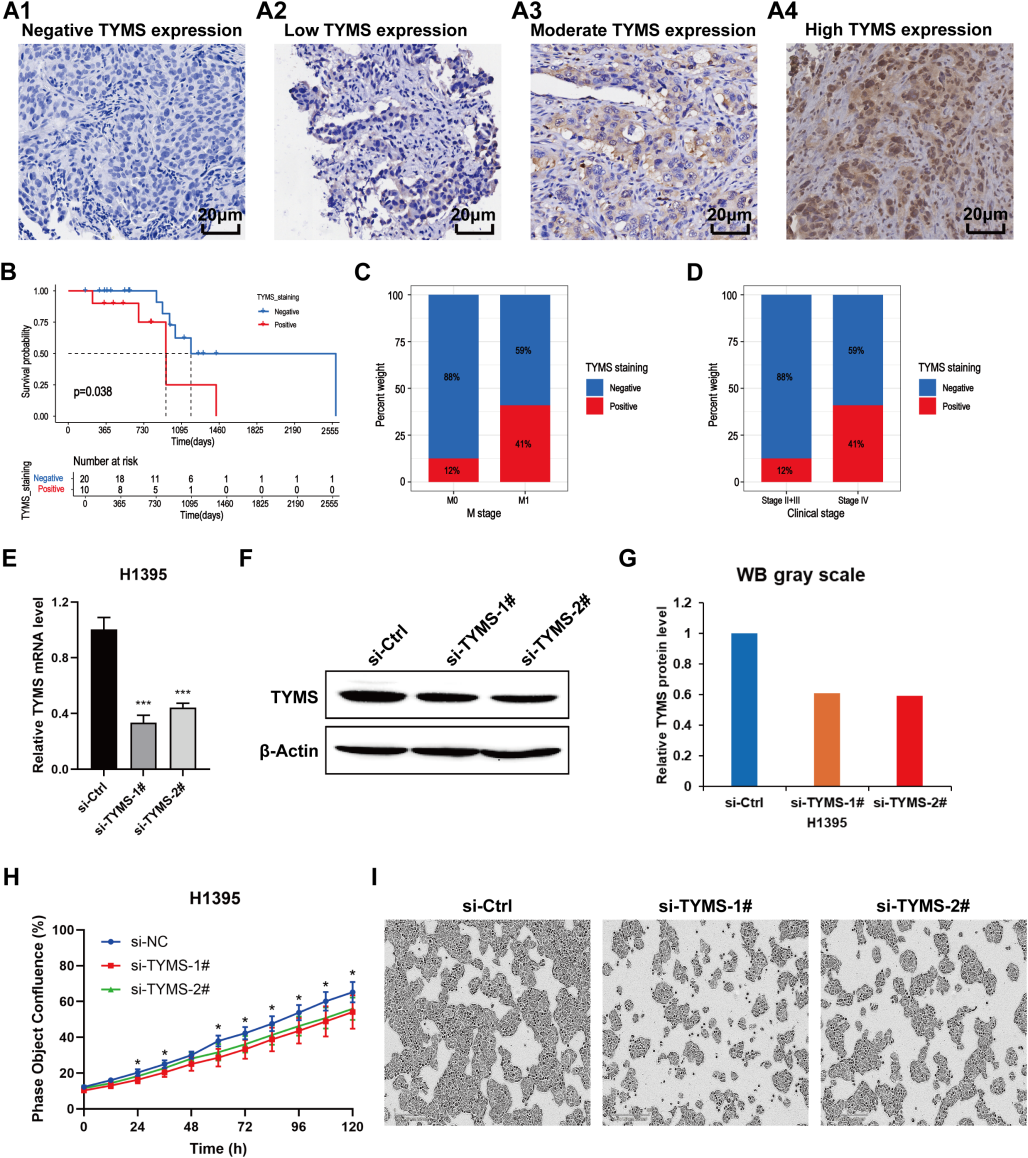
Validation of the clinical and biological roles of *TYMS* through experiments on LUAD clinical samples (n=30) and cell lines. **(A)** Images of LUAD samples with negative *TYMS* expression **(A1)**, low *TYMS* expression **(A2)**, moderate *TYMS* expression **(A3)** and high *TYMS* expression **(A4)** in the immunohistochemical experiment. **(B)** Kaplan–Meier curves showing the survival status of LUAD patients with negative and positive *TYMS* staining. **(C)** Percentage plot showing the proportion of samples with negative and positive staining among tumors at the M0 and M1 stages. **(D)** Percentage plot showing the proportions of samples with negative and positive staining among patients with clinical stages II+III and clinical stage IV disease. **(E)** Column chart showing the relative mRNA expression levels of *TYMS* in the si-*TYMS*-1#, si-*TYMS*-2# and control groups via qRT–PCR. **(F)** Western blotting results showing the protein levels of *TYMS* in the si-*TYMS*-1#, si-*TYMS*-2# and control groups. **(G)** The corresponding grayscale of the WB results in **(F)**. **(H)** Proliferation curves of H1395 cells in the si-*TYMS*-1#, si-*TYMS*-2# and control groups. **(I)** Images of H1395 cells in the si-*TYMS*-1#, si-*TYMS*-2# and control groups captured by the IncuCyte platform 120 hours after seeding into 96-well plates.

Previous results revealed that *TYMS* was a hub gene in the T_RM_ cell-related prognostic signature in the PPI analysis and the construction of the risk score model (**Figures 2D**, **3E**; **Supplementary Table 3**). However, the function of *TYMS* in LUAD has rarely been investigated. To further investigate the clinical and biological roles of *TYMS* in LUAD, we performed immunohistochemical staining experiments using LUAD samples and assessed the proliferation of LUAD cell lines. Images of LUAD samples with negative, low, moderate, and high *TYMS* expression, magnified 40 × 10 times under a light microscope, are presented in (**Figures 11A1–A4**). The clinical samples included in this study were from a total of 30 patients with LUAD, 10 of whom were positive for *TYMS* and 20 of whom were negative. Survival analysis revealed that patients with negative *TYMS* staining in their tumor tissues had longer overall survival times than patients with positive *TYMS* staining (p=0.038, **Figure 11B**). Patients with M1 tumors had a higher *TYMS*-positive staining rate than those with M0 tumors (**Figure 11C**), and patients with clinical stage IV tumors had a higher *TYMS*-positive staining rate than those with stages II and III tumors (**Figure 11D**). These findings indicated that *TYMS* is a risk factor for LUAD patients, which is consistent with our previous findings (**Figure 3E**; **Supplementary Table 3**).

In the cell proliferation experiment, first, *TYMS* was successfully knocked down through the siRNA oligos, as evaluated by qRT-PCR (**Figure 11E**) and western blotting (**Figures 11F, G**). The results of long-term dynamic observation experiments using the IncuCyte platform revealed that the proliferative capacity of H1395 cells was significantly impaired in the *TYMS*-knockdown groups compared with the control groups (**Figures 11H, I**), suggesting that *TYMS* may promote LUAD cell proliferation. These findings validate the role of *TYMS* in enhancing the growth of H1395 cells, suggesting its potential in promoting LUAD progression.

### 3.9 Validating the correlation between the T_RM_ cells infiltration density and the expression levels of the prognostic predictors in LUAD

**Figure 12:**
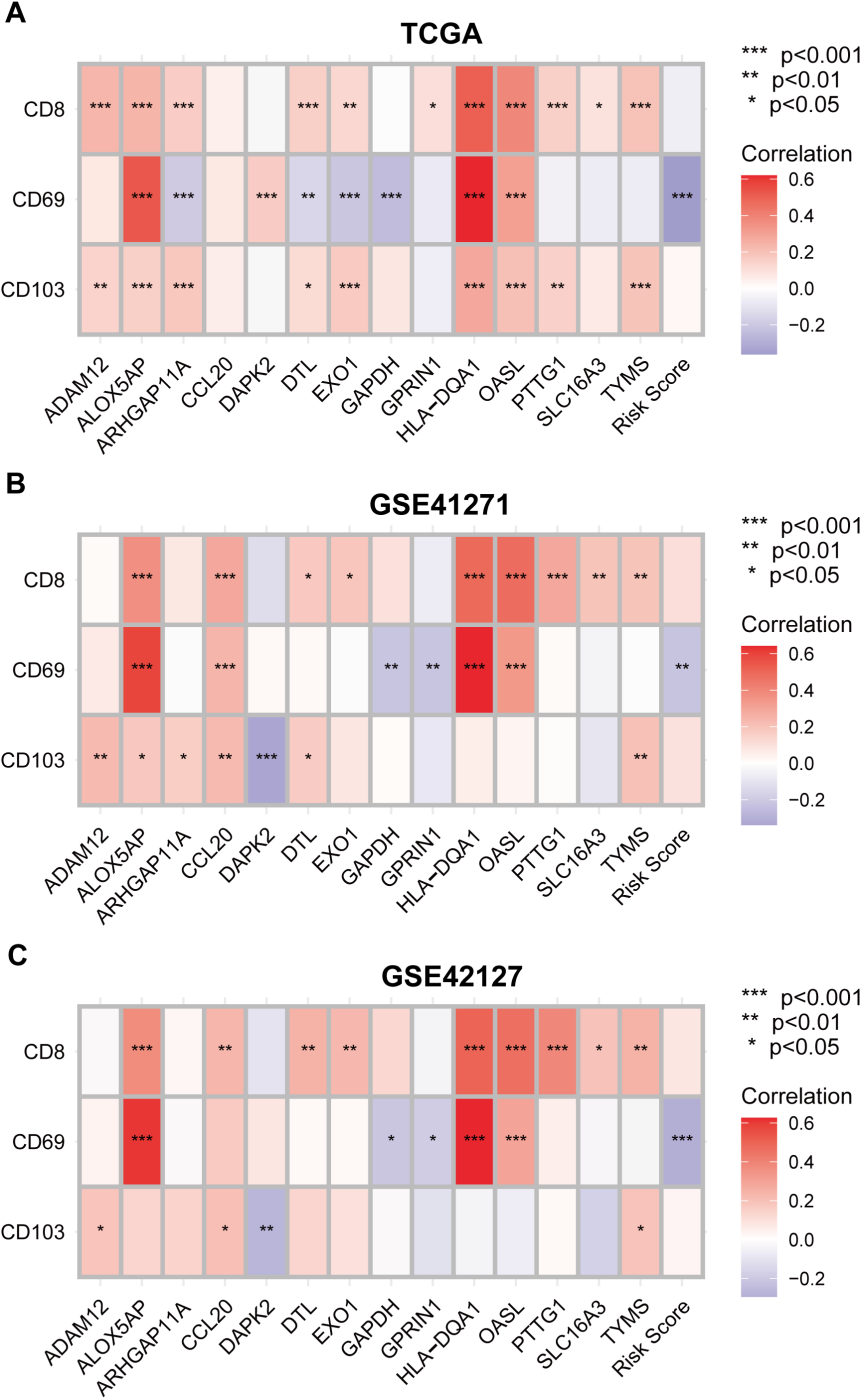
Validating the correlation between the tissue-resident memory T cells infiltration density and the expression levels of the prognostic predictors in LUAD. **(A–C)** Correlation analysis between T_RM_ cell marker genes *CD8*, *CD69* and *CD103*, and the 14 genes involved in the T_RM_ cell-related prognostic signature using TCGA **(A)**, GSE 41271 **(B)** and GSE42127 **(C)** datasets. Negative correlation: blue; positive correlation: red.

To further verify the link between tissue-resident memory T cells infiltration and the expression of the T_RM_ cell-related prognostic predictors, Spearman’s correlation analysis was performed between the expression levels of the marker genes of T_RM_ cells: *CD8*, *CD69*, *CD103*, and the 14 genes included in the T_RM_ cell-related prognostic signature. The analytical results were consistent in the three datasets: TCGA (**Figure 12A**), GSE41271 (**Figure 12B**), and GSE42127 (**Figure 12C**). Interestingly, the expression levels of genes that were significant risk factors for LUAD patient prognosis, such as *GAPDH*, *GPRIN1* and *EXO1*, were negatively correlated with the expression levels of T_RM_ cell marker genes (**Figures 3E**, **12A–C**). In contrast, the genes that are protective factors for the prognosis of LUAD, such as *HLA-DQA1* and *ALOX5AP*, were positively correlated with the three T_RM_ cell markers (**Figures 3E**, **12A–C**). Moreover, the risk score of the LUAD patients was negatively correlated with the important T_RM_ cell marker gene *CD69* in all three cohorts (**Figures 12A–C**). These results confirmed again that the prognostic predictors involved in the T_RM_ cell-related signature could affect patient prognosis by regulating the infiltration of T_RM_ cells.

## 4 Discussion

T_RM_ cells can reside in specific organs or tissues without entering the blood circulation (1). A recent study revealed that T_RM_ cells in different tissues or organs have distinct transcriptomic status and specific gene expression patterns, which may be closely related to their specific functions in these organs (11). For example, acute respiratory virus-specific T_RM_ cells, such as influenza- and SARS-CoV-2-specific T_RM_ cells, are more likely to be maintained in the lungs than at other sites; T_RM_ cells that target the intestinal microbiota and pathogens are maintained in the intestinal tract, whereas in the skin, T_RM_ cells are produced in response to skin infections and are associated with protective immune responses. The infiltration of T_RM_ cells has been proven to be a protective factor for patient prognosis in many cancers (10). Recent studies have shown that even the dysfunctional CD8^+^T_RM_ cells, induced by interactions with surrounding tumor cells, play important roles in anti-tumoral reactivity (23, 24). The efficacy of anti-PD-1 immunotherapy is also highly dependent on the sufficiency of CD8^+^T_RM_ cells infiltrating in the TME (25). However, it is unclear whether T_RM_-specific genes in one type of organ can regulate T_RM_ cells to exert specific immune responses against tumors.

To explore the roles of T_RM_-specific genes in regulating the development of LUAD, in this study, T_RM_ cell-specific genes that were differentially expressed (DE) between LUAD and adjacent normal tissues were identified (**Figures 2A–D**; **Supplementary Tables 1**, **2**). Then, 62 T_RM_-specific DE genes associated with OS were identified through univariate Cox regression analysis (**Supplementary Table 3**), and a T_RM_-related prognostic model was constructed based on these genes (**Figures 3A–E**). The prognostic model showed strong predictive efficacy in both the training and validation cohorts (**Figures 4A–I**). Patients with low- and high-risk scores also had different clinical features according to the prognostic model (**Supplementary Figures 1**, **2**). Fourteen lung T_RM_ cell-specific genes were included in the prognostic model (**Figure 3E**). Among the 14 genes, *GAPDH*, *SLC16A3*, *PRIN1*, *TYMS*, *CCL20*, *ARHGAP11A*, *DTL*, *PTTG1*, *EXO1*, *OASL* and *ADAM12* were risk factors, whereas *DAPK2*, *ALOX5AP* and *HLA-DQA1* were protective factors for the prognosis of patients with LUAD (**Figure 3E**). Our results are consistent with those of previously published studies (26–38). We noticed that *TYMS* and *EXO1* had strong connectivity with other T_RM_ cell-specific genes in the PPI analysis; therefore, these two genes may play central roles in the regulatory network of these genes (**Figure 2D**). We intend to further explore the clinical and biological functions of these two genes in our future studies, and our research on the functions of *EXO1* in LUAD has recently been published recently (39).

The results of the GSEA of the DEGs between the low- and high-risk score groups revealed that some pathways related to the antitumoral response, such as the B-cell receptor signaling pathway (40), regulation of T-cell activation and T-cell-mediated immunity (41), were activated in the low-risk score group (**Figures 5A, B**; **Supplementary Table 4**). Some immune cells that play important roles in killing tumor cells also infiltrated more in the low-risk score group, such as activated CD8^+^T cells and central memory CD8 T cell (22, 23, 41, 42) (**Figure 5C**). This was validated by correlation analysis between CD8^+^T_RM_ cell marker genes *CD8*, *CD69*, *CD103* (2), the T_RM_ cell-related risk score, and the 14 genes involved in the T_RM_ cell-related prognostic signature. Among the 14 genes, genes that are associated with inferior prognosis of LUAD patients, such as *GAPDH*, *GPRIN1* and *EXO1*, were negatively correlated with the expression levels of T_RM_ cells markers (**Figures 3E**, **12A–C**). However, genes that were associated with superior prognosis of LUAD patients, such as *HLA-DQA1* and *ALOX5AP*, were positively correlated with T_RM_ cell marker genes (**Figures 3E**, **12A–C**). The risk score, which was associated with poor prognosis, was significantly negatively correlated with the T_RM_ cell marker *CD69* in all three cohorts (**Figures 4**, **12**). This confirmed that the T_RM_ cell-related risk score was negatively associated with T_RM_ cell infiltration in LUAD, and the prognostic predictors involved in the risk score model may impact the prognosis of LUAD patients by regulating T_RM_ cell infiltration. These findings suggest that the risk score was negatively correlated with antitumor immune activation in LUAD patients, which may be an important reason for the better prognosis in the low-risk score group. Moreover, most of the reported immune checkpoint genes were highly expressed in the low-risk score group (**Figure 5E**). This finding was consistent with the finding that patients in the low-risk score group had better clinical outcomes after treatment with immune checkpoint blockade (**Figures 8C–F**).

Given that single-cell sequencing is an efficient method for studying the spatiotemporal heterogeneity of tumors (43–45), we further analyzed the scRNA-seq data to validate the predictive efficacy of the T_RM_-related prognostic model (**Supplementary Figures 3**, **4**). Three subtypes of malignant cells were identified, namely, *IFI27* ^+^Mal, *FBXO2* ^+^Mal and *HMGB2* ^+^Mal (**Figures 6A–C**). The results also revealed that the *FBXO2* ^+^Mal and *HMGB2* ^+^Mal cell subtypes had significantly higher scores than *IFI27* ^+^Mal cells (**Figures 6D–G**). Functional enrichment analysis revealed that the marker genes of *IFI27* ^+^Mal cells were enriched in pathways related to immune activation, such as the positive regulation of leukocyte and MHC class II receptor activity (**Supplementary Figure 5**; **Supplementary Table 5**), which was consistent with previous findings that the T_RM_-related risk score and cell score were negatively correlated with immune activation (**Figures 5A–D**; **Supplementary Figure 6A**). *FBXO2*, *HMGB2* and *IFI27* are all oncogenes according to previous reports (46–51), and their roles in regulating the development of LUAD and the possibility of becoming therapeutic targets need to be further studied.

The above results were mainly based on analyses of RNA expression data. In subsequent analyses, the protein expression levels of the genes included in the T_RM_-related signature were investigated using immunohistochemical images from the Human Protein Atlas database (https://www.proteinatlas.org/). *SLC16A3*, *ARHGAP11A*, *PTTG1*, *GPRIN1* and *TYMS* were more highly expressed in LUAD tissues, whereas *HLA-DQA1*, *ALOX5AP* and *OASL* were expressed at lower levels in LUAD tissues than in normal lung tissues. These findings validated the differences in the RNA expression of these genes (**Figures 9A–J**). Survival analysis via proteomic data verified the prognostic significance of several T_RM_-related signature genes, including *SLC16A3*, *TYMS*, *HLA-DQA1*, *ALOX5AP* and *OASL* (**Figures 10A–E**). Among these genes included in the T_RM_-related signature, *TYMS* appeared to be a hub gene. *TYMS* has been reported to be an oncogene for colorectal cancer, pancreatic cancer and lymphoma and promotes tumor progression (52–54), but its role in LUAD has rarely been reported. Our experiments using LUAD clinical samples and cell lines demonstrated that *TYMS* could also promote the progression of LUAD (**Figures 11A–I**), and may be a potential therapeutic target that could regulate the functions of T_RM_ cells in LUAD. A few reports have suggested that the expression levels of *TYMS* are associated with the TME landscape and responses to immune checkpoint inhibitor therapy (55, 56). However, the underlying mechanisms have not been elucidated. *TYMS* is a key enzyme in the 5-FU catabolic pathway and is associated with the response to 5-FU-based therapy (57). A study has also demonstrated that 5-FU/platinum chemotherapy could facilitate tumor-reactive T cell and M1-like macrophage interactions, thus improving the efficacy of anti-PD-1 immunotherapy for advanced gastroesophageal adenocarcinomas (GEA) (58). Therefore, *TYMS* may regulate the TME and anti-PD-1 immunotherapeutic response via its roles in 5-FU metabolism. Moreover, *TYMS* is regulated by *MYC*, which affects *PD-1* expression in colorectal cancer (59). Thus, *TYMS* may also affect anti-PD1 immunotherapy through *MYC*.

Our study had some limitations. First, our study was based mainly on bioinformatics analyses of public datasets and was only partially verified by experiments on clinical tissues. Biological and molecular experiments *in vitro* and/or *in vivo* are needed to further investigate the functions of key genes and the activities of the corresponding signaling pathways. Second, owing to the retrospective nature of our study, bias may be inevitable, and prospective experiments are needed for further validation. Third, the differentially expressed T_RM_ cell-related genes were identified with thresholds set at |log2FC|≥1 and FDR<0.05, using bioinformatic methods. Although they are standard methods, some genes that do not meet with the threshold criteria may also play significant roles in the functions of lung T_RM_ cells and the development of LUAD. Moreover, the experimental validation in our study focused primarily on the PPI hub gene *TYMS* owing to the limitation of time and experimental conditions. In the future studies, we will extend these functional studies to other key genes.

## 5 Conclusion

The prognostic signature based on lung T_RM_ cell-related genes was efficient and robust for predicting the prognosis and therapeutic outcomes of patients with LUAD. The expression and functions of key genes in the prognostic signature were verified through experiments with LUAD samples and cell lines. Our findings increase the understanding of T_RM_-related clinical and biological significance in LUAD and may provide potential therapeutic targets for LUAD.

## Data Availability

The raw data supporting the conclusions of this article will be made available by the authors, without undue reservation.

## Supplementary material

Supplementary Figure 1 The percentages of patients with different ages **(A)**, tumor stages **(B)**, sexes **(C)**, *EGFR* alteration status **(D)**, *ALK-EML4* fusion status **(E)**, *KRAS* alteration status **(F)** and smoking history **(G)** in the low- and high-risk score groups.

Supplementary Figure 2 Box plots showing the risk scores of patients with different ages **(A)**, tumor stages **(B)**, sexes **(C)**, *EGFR* alteration status **(D)**, smoking history **(E)** and *KRAS* alteration status **(F)**.

Supplementary Figure 3 Quality control of the scRNA-seq data. **(A–F)** Correlation analysis between nFeature_RNA and nCount_RNA **(A)**, percent.mito and nCount_RNA **(B)**, percent.HB and nCount_RNA **(C)**, percent.mito and nFeature_RNA **(D)**, percent.HB and nFeature_RNA **(E)**, percent.HB and percent.mito **(F)**. The correlation coefficients were marked on the top of each panal. **(G–J)** The nFeature_RNA **(G)**, nCount_RNA **(H)**, percent.mito **(I)** and percent.HB **(J)** in different LUAD samples. The abscissa axes show the names of LUAD samples, whilst the ordinate axes show the numbers or percentages of each items.

Supplementary Figure 4 Annotation of the cell types in the scRNA-seq data. **(A)** ElbowPlot of the PCA. **(B)** PCA of the scRNA-seq data. **(C)** The distribution of cells before the removal of the batch effect. **(D)** The distribution of cells after the removal of the batch effect. **(E)** The TSNE plot showing the distribution of the cell types annotated in the harmony analysis. Different colors represent different cell types. The names of the cell types are annotated on the right of the plot. **(F)** The percent bar chart showing the proportions of different types of cells in the LUAD tissues. The abscissa axes show the names of LUAD samples, whilst the ordinate axes show the percentage weight of each cell. The figure note is marked on the right.

Supplementary Figure 5 Functional enrichment analysis for the marker genes of *FBXO2* ^+^ **(A1–A4)**, *HMGB2* ^+^ **(B1–B4)** and *IFI27* ^+^ **(C1–C4)** malignant tumor cells.

Supplementary Figure 6 GSEA of the DEGs between the low- and high-CellScore groups. **(A)** Pathways that are activated in different risk score groups according to KEGG enrichment analysis of the scRNA-seq data. **(B)** Pathways that are activated in different risk score groups according to the results of the GO-BP enrichment analysis of the scRNA-seq data. The abscissa axis represents the list of genes ranked according to their expression levels in the two groups. The vertical axis represents the running enrichment score. Curves of different colors represent different pathways.

Supplementary Figure 7 Cellular communication analysis for the high- and low-CellScore groups. **(A)** The number of receptor–ligand pairs between different cell populations. The sizes of the dots represent the number of corresponding cells. The thickness of the lines indicates the number of receptors and ligands between different cell populations. The color of the connecting line was the same as the color of the signal emitter. **(B)** Statistical dot plot of the dominant signaling pathways. The colors of the dots indicate different cell populations. The sizes of the dots are proportional to the number of ligands and receptors inferred from each cell population, and the x- and y-axes indicate the strengths of the cell populations as signal senders and receivers, respectively. **(C)** Statistical heatmap of the signaling dominant of the significant pathways. The abscissa axis indicates the cell, and the vertical axis indicates the names of the signaling pathways. **(D)** Dot plot of the signaling dominant of the significant pathways. The abscissa axis indicates the names of the signaling pathways, and the vertical axis indicates the cell names. **(E)** Tumor cells in the high-CellScore group had stronger cellular communication with myeloid cells in the CSF signaling pathway network. **(F)** Tumor cells in the high-CellScore group had stronger cellular communication with mast cells in the KIT signaling pathway network.

Supplementary Table 1 Differential expression analysis of genes between LUAD and adjacent normal tissues.

Supplementary Table 2 The intersecting genes of the DEGs between LUAD and adjacent normal tissues and between lung T_RM_ cell-related genes.

Supplementary Table 3 The 62 prognostic genes identified in the univariate Cox regression analysis.

Supplementary Table 4 KEGG GSEA and GO-BP GSEA enrichment analysis of the DEGs between the low- and high-risk score groups in the bulk RNA-seq data.

Supplementary Table 5 The marker genes of each subtype of malignant cells.

Supplementary Table 6 KEGG GSEA and GO-BP GSEA enrichment analysis of the DEGs between the low- and high-risk score groups in the scRNA-seq data.

Supplementary Table 7 Correlation analysis between the risk score and the log2(IC50) values of the drugs.
