# Supplementary figures and images for: Revealing the significance of tissue-resident memory T cells in lung adenocarcinoma through bioinformatic analysis and experimental validation
Source: Front Immunol. 2025 Jun 26;16:1600863. doi: 10.3389/fimmu.2025.1600863 (PMC12240778; doi:10.3389/fimmu.2025.1600863)

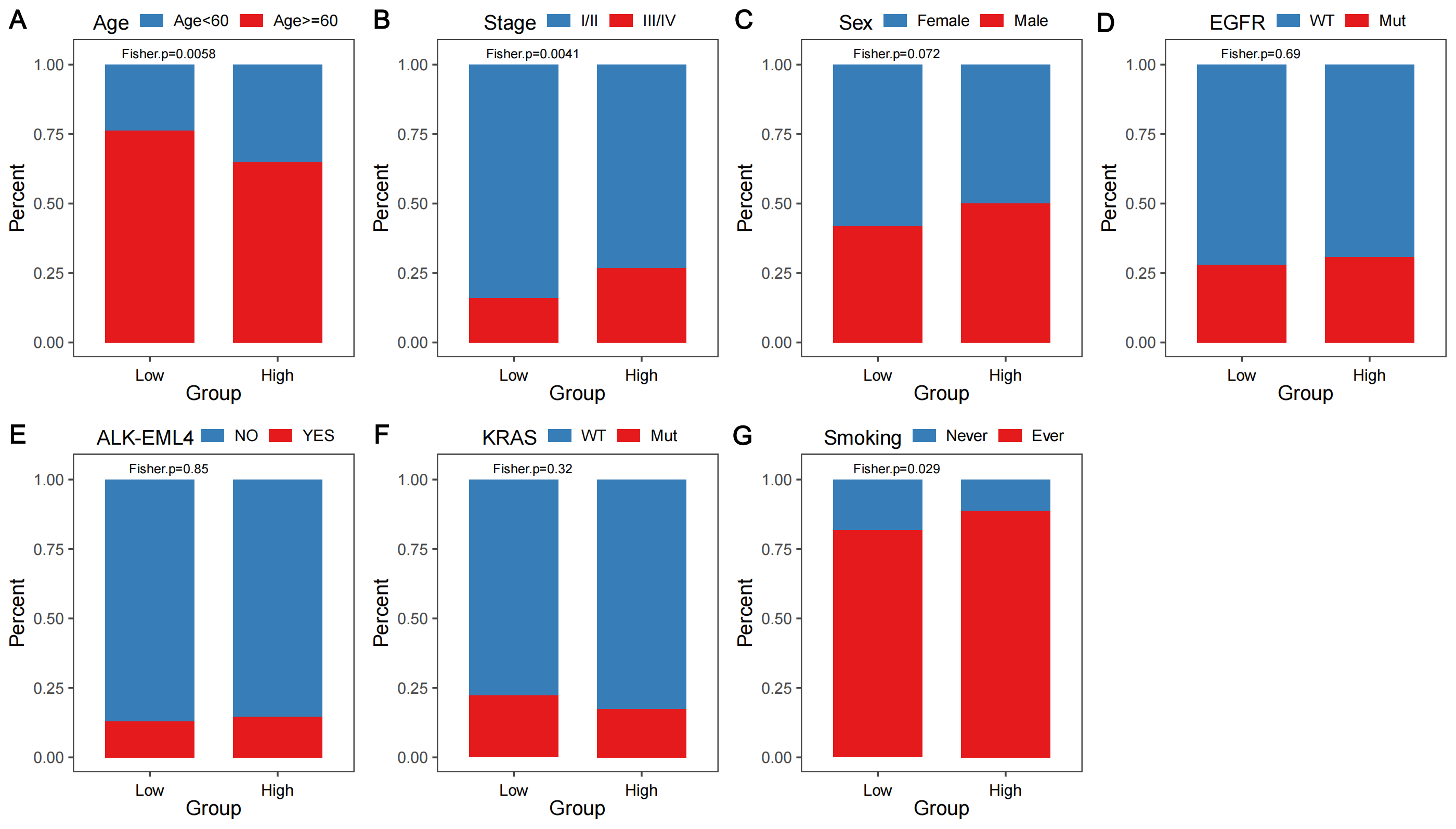

Supplement: Supplementary Figure 1 — The percentages of patients with different ages (A), tumor stages (B), sexes (C), EGFR alteration status (D), ALK-EML4 fusion status (E), KRAS alteration status (F) and smoking history (G) in the low- and high-risk score groups. [file Image1.tif]

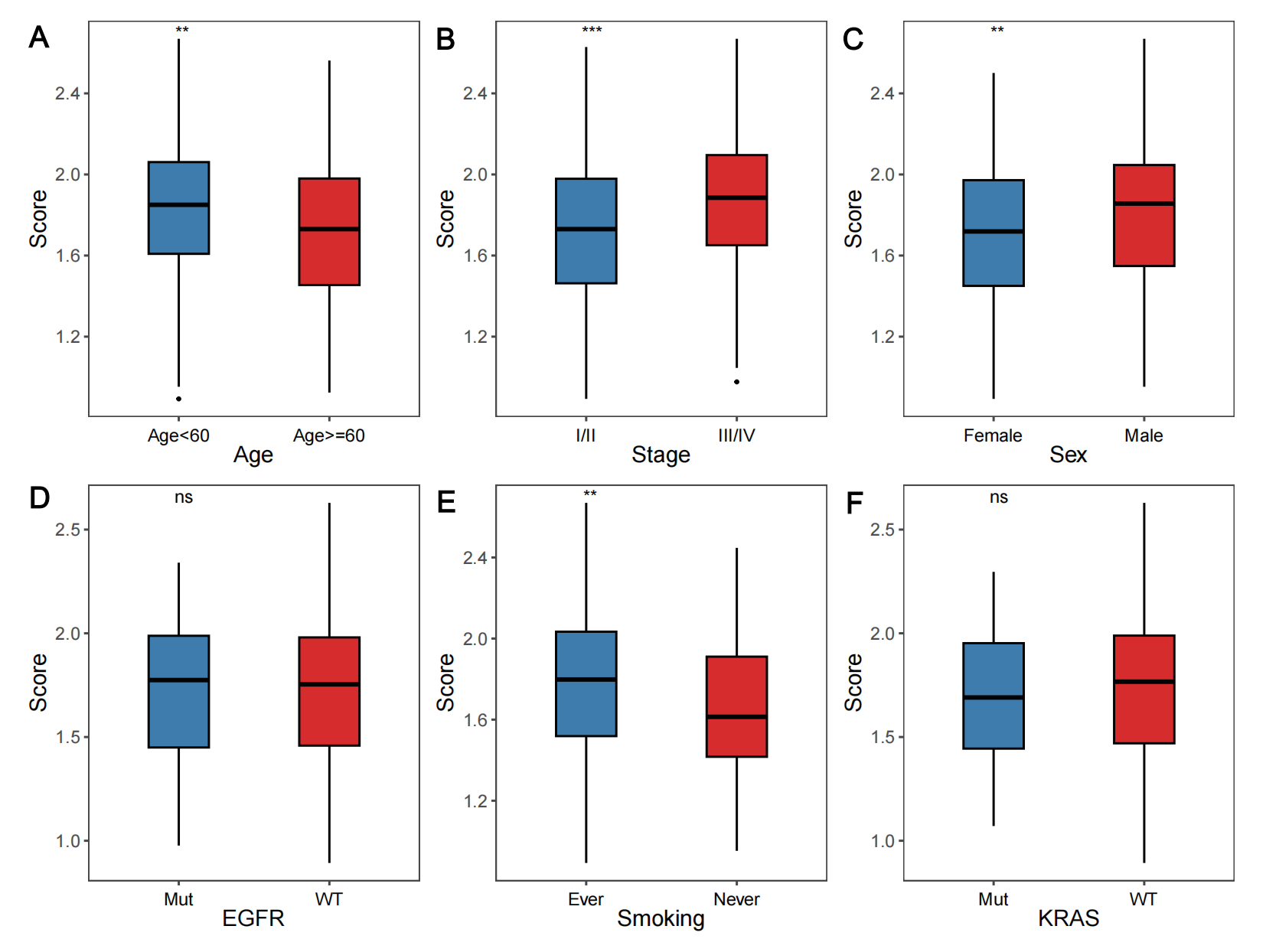

Supplement: Supplementary Figure 2 — Box plots showing the risk scores of patients with different ages (A), tumor stages (B), sexes (C), EGFR alteration status (D), smoking history (E) and KRAS alteration status (F). [file Image2.tif]

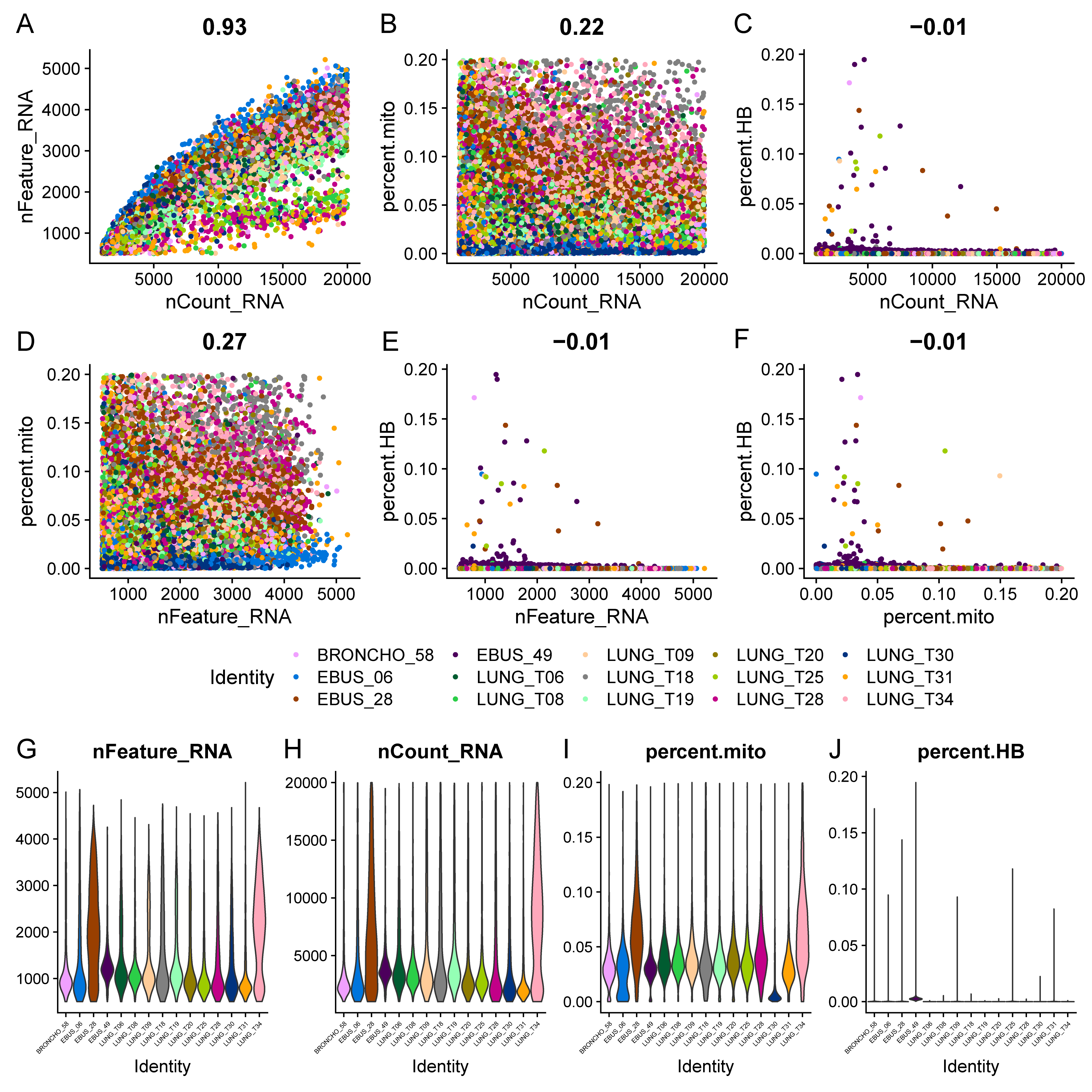

Supplement: Supplementary Figure 3 — Quality control of the scRNA-seq data. (A–F) Correlation analysis between nFeature_RNA and nCount_RNA (A), percent.mito and nCount_RNA (B), percent.HB and nCount_RNA (C), percent.mito and nFeature_RNA (D), percent.HB and nFeature_RNA (E), percent.HB and percent.mito (F). The correlation coefficients were marked on the top of each panal. (G–J) The nFeature_RNA (G), nCount_RNA (H), percent.mito (I) and percent.HB (J) in different LUAD samples. The abscissa axes show the names of LUAD samples, whilst the ordinate axes show the numbers or percentages of each items. [file Image3.tif]

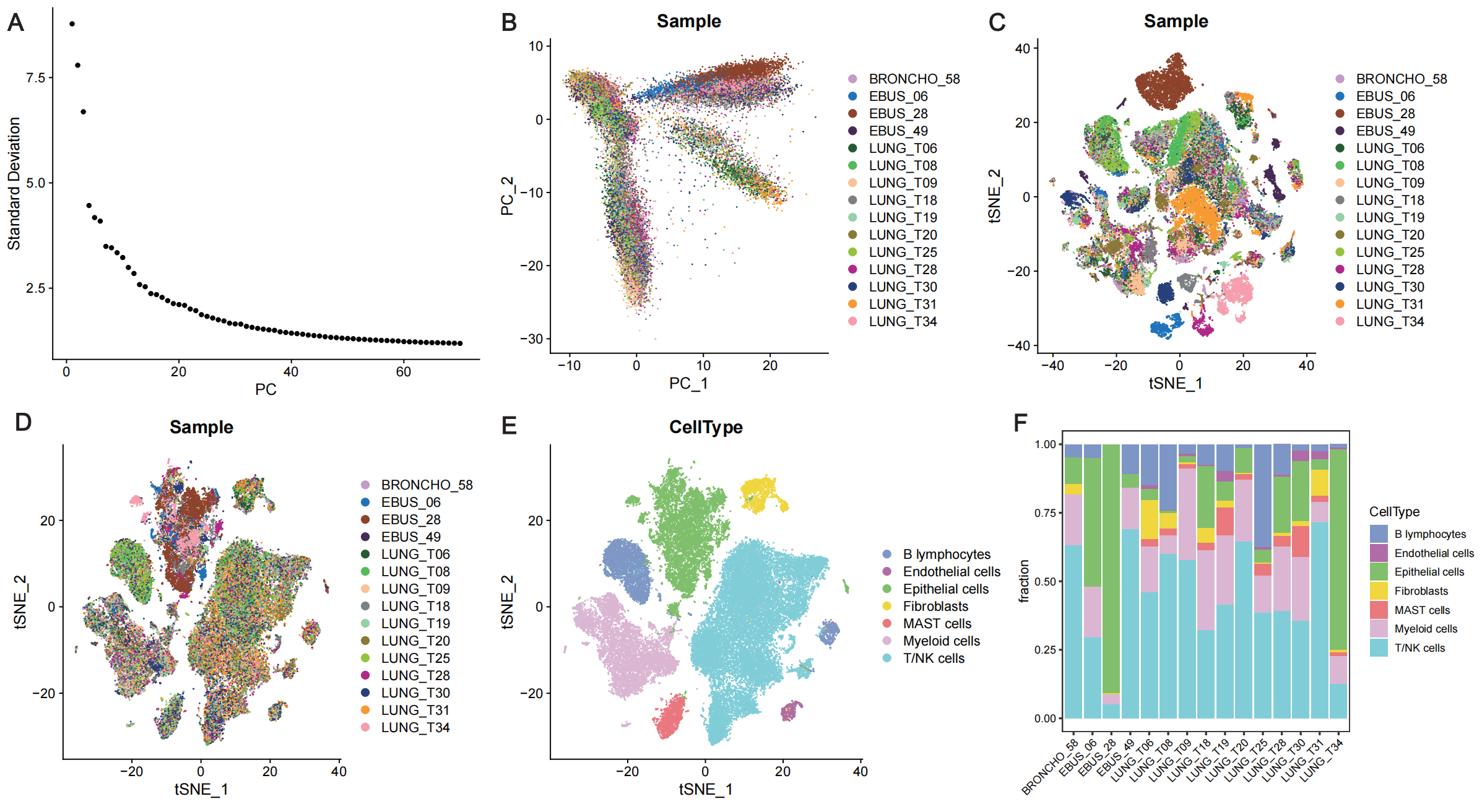

Supplement: Supplementary Figure 4 — Annotation of the cell types in the scRNA-seq data. (A) ElbowPlot of the PCA. (B) PCA of the scRNA-seq data. (C) The distribution of cells before the removal of the batch effect. (D) The distribution of cells after the removal of the batch effect. (E) The TSNE plot showing the distribution of the cell types annotated in the harmony analysis. Different colors represent different cell types. The names of the cell types are annotated on the right of the plot. (F) The percent bar chart showing the proportions of different types of cells in the LUAD tissues. The abscissa axes show the names of LUAD samples, whilst the ordinate axes show the percentage weight of each cell. The figure note is marked on the right. [file Image4.tif]

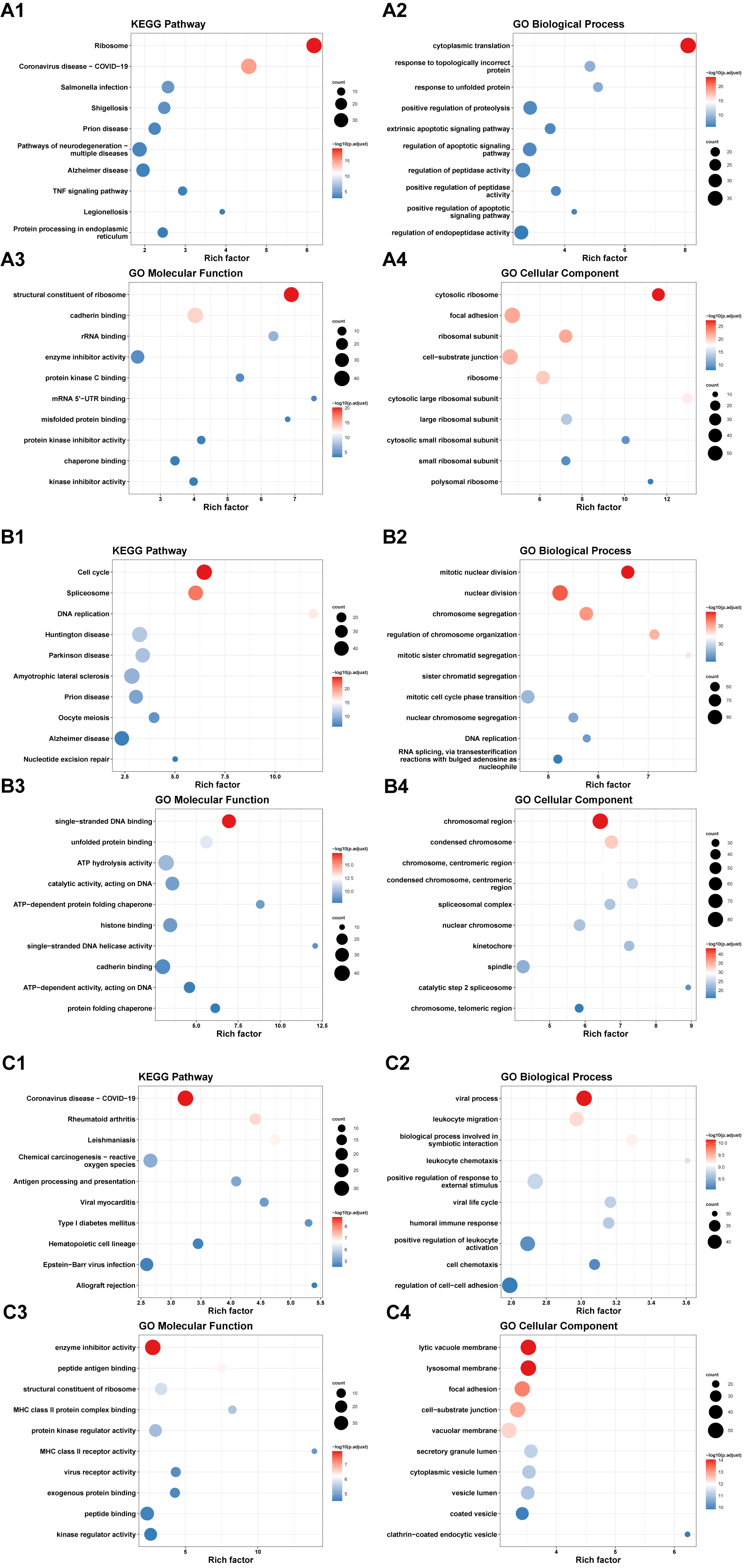

Supplement: Supplementary Figure 5 — Functional enrichment analysis for the marker genes of FBXO2 + (A1–A4), HMGB2 + (B1–B4) and IFI27 + (C1–C4) malignant tumor cells. [file Image5.tif]

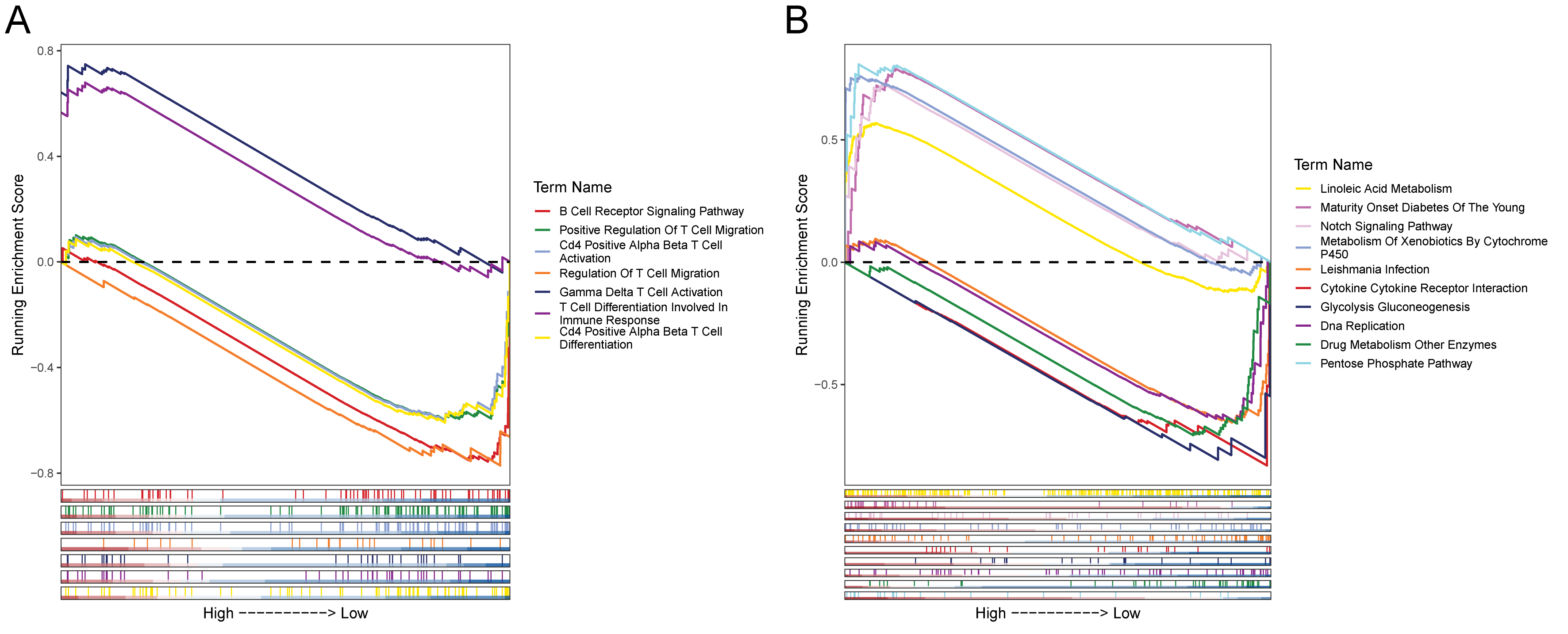

Supplement: Supplementary Figure 6 — GSEA of the DEGs between the low- and high-CellScore groups. (A) Pathways that are activated in different risk score groups according to KEGG enrichment analysis of the scRNA-seq data. (B) Pathways that are activated in different risk score groups according to the results of the GO-BP enrichment analysis of the scRNA-seq data. The abscissa axis represents the list of genes ranked according to their expression levels in the two groups. The vertical axis represents the running enrichment score. Curves of different colors represent different pathways. [file Image6.tif]

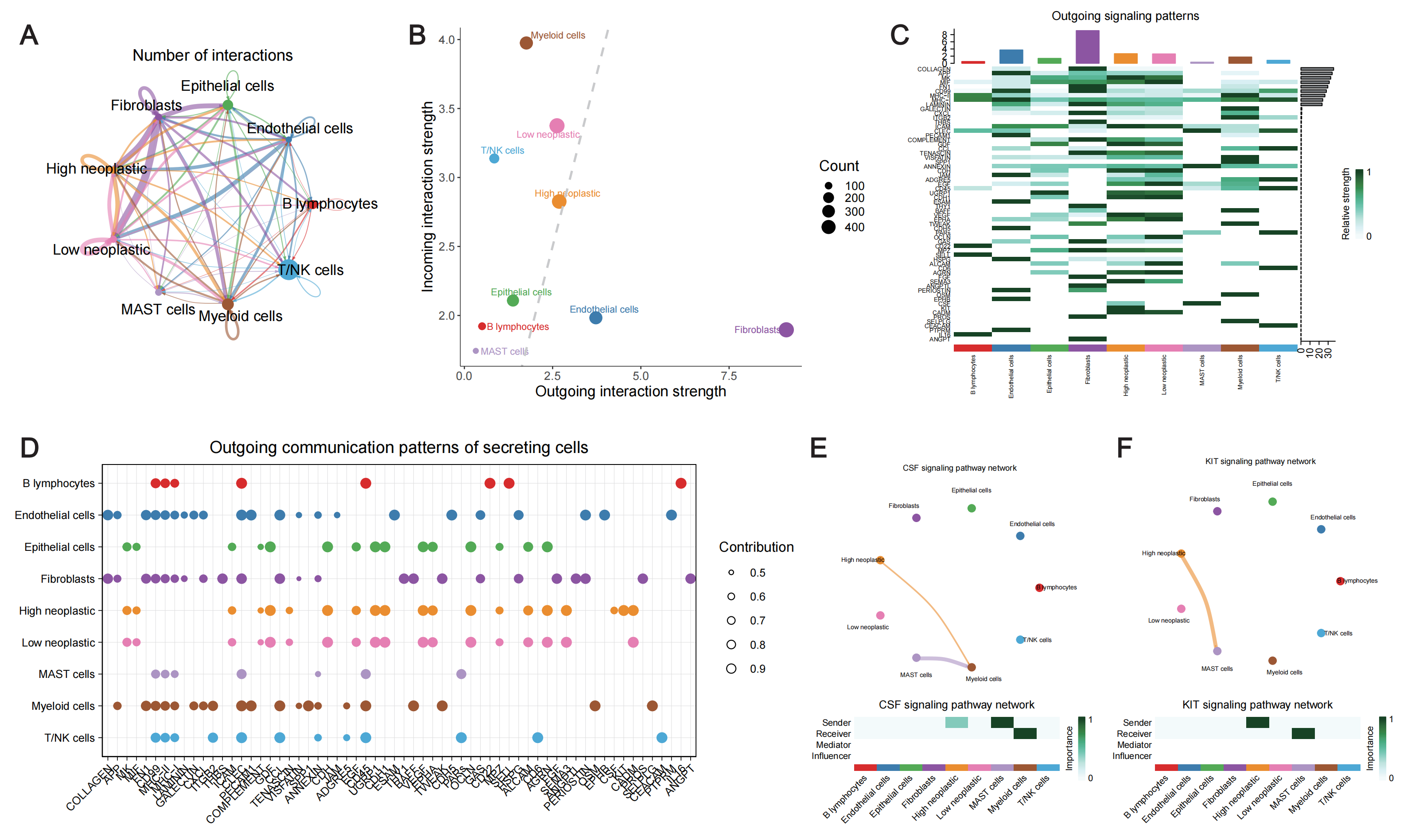

Supplement: Supplementary Figure 7 — Cellular communication analysis for the high- and low-CellScore groups. (A) The number of receptor–ligand pairs between different cell populations. The sizes of the dots represent the number of corresponding cells. The thickness of the lines indicates the number of receptors and ligands between different cell populations. The color of the connecting line was the same as the color of the signal emitter. (B) Statistical dot plot of the dominant signaling pathways. The colors of the dots indicate different cell populations. The sizes of the dots are proportional to the number of ligands and receptors inferred from each cell population, and the x- and y-axes indicate the strengths of the cell populations as signal senders and receivers, respectively. (C) Statistical heatmap of the signaling dominant of the significant pathways. The abscissa axis indicates the cell, and the vertical axis indicates the names of the signaling pathways. (D) Dot plot of the signaling dominant of the significant pathways. The abscissa axis indicates the names of the signaling pathways, and the vertical axis indicates the cell names. (E) Tumor cells in the high-CellScore group had stronger cellular communication with myeloid cells in the CSF signaling pathway network. (F) Tumor cells in the high-CellScore group had stronger cellular communication with mast cells in the KIT signaling pathway network. [file Image7.tif]
